# From Fluorinated to All-Fluorine-Free Systems: Hückel Anion-Based Electrolytes for Lithium–Sulfur Batteries with Enhanced High C‑Rate Sulfur Utilization

**DOI:** 10.1021/acsomega.6c01071

**Published:** 2026-06-17

**Authors:** Maciej Smoliński, Adam Łaszcz, Aleksandra Ossowska, Marek Marcinek, Maciej Marczewski

**Affiliations:** † 49566 Warsaw University of Technology, Faculty of Chemistry, Noakowskiego 3, Warsaw 00-664, Poland; ‡ Łukasiewicz Research Network, Institute of Microelectronics and Photonics, al. Lotników 32/46, Warsaw 02-668, Poland

## Abstract

Lithium–sulfur (Li–S) batteries are considered a promising alternative to conventional lithium-ion systems; however, their practical deployment is still limited by insufficient sulfur utilization, polysulfide shuttling, and capacity fading. In this work, two complementary modification strategies for Li–S batteries are systematically investigated. First, obtaining simple, easy-to-prepare, and low-cost model cathodes enables the simulation of cycling of the cell with different sulfur availability through a comparative assessment of conductive carbon blacks (Super P, Ketjen Black EC-300-JD, and Vulcan XC-72), NaCMC binder, and sulfur content. The influence of electrode formulation, sulfur loading, electrolyte-to-sulfur (E/S) ratio, and conductive carbon type on electrochemical performance is evaluated, enabling the identification of an optimized model cathode. In the second part of the study, electrolyte optimization is explored using a series of Hückel anion-based lithium salts, including fluorine-free candidates. The electrochemical performance of lithium 4,5-dicyano-2-(n-heptafluoropropyl)­imidazolide (LiHDI), lithium 4,5-dicyano-2-(pentafluoroethyl)­imidazolide (LiPDI), lithium 4,5-dicyano-2-(trifluoromethyl)­imidazolide (LiTDI), and lithium 2,4,5-tricyanoimidazolide (LiTIM) is benchmarked against the conventional LiTFSI-based electrolyte. The investigated salts generally deliver higher discharge capacities, particularly at elevated charge rates, and exhibit improved capacity retention during prolonged cycling. The combined cathode and electrolyte engineering approach demonstrates enhanced electrochemical stability and reduced capacity decay, indicating that Hückel anion-based electrolytes represent a viable direction for further optimization of Li–S battery systems.

## Graphical abstract

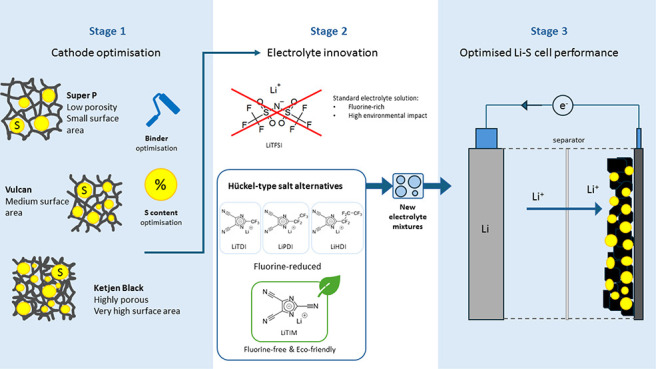

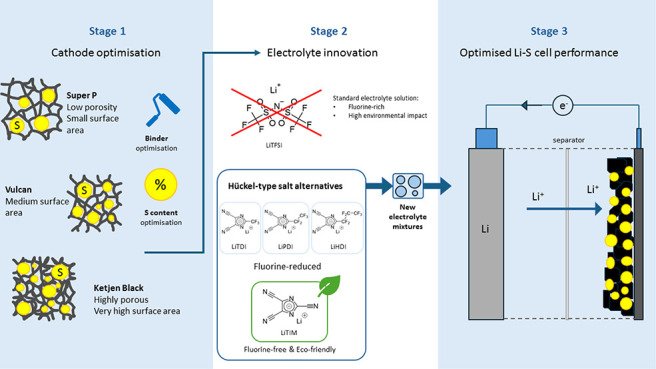

## 1 Introduction

For many years now, it can be observed that, along with the developing world, human demand for energy is increasing. In the early twenties of the 21st century, having electric (EV) and hybrid (HEV) vehicles, computers, or cell phones—a good-performing battery is the thing that everybody expects.^1–3^ Lithium-sulfur batteries are rapidly gaining interest as a promising successor to the commercial cells that are produced now.^4–6^ Due to their high theoretical specific capacity (1675 mAh g^–1^) and high energy density (2600 Wh kg^–1^)^7^ they seem to be a good successor to commercial and widely spread lithium-ion batteries. Also, elementary sulfur used in the cathode is more environmentally friendly^8,9^ and much easier to acquire than the transition metal oxides used in lithium-ion technology.^10^

Sulfur atoms exhibit a strong tendency to form homoatomic chains of various lengths.^11^ One of the most stable allotropes of sulfur at room temperature is orthorhombic sulfur (α-sulfur).^12^ During the discharge process, the redox reaction on the cathode can be presented in a simplified way as 16Li^+^ + S_8_ + 16e^–^ = 8Li_2_S occurs.^13^ In reality, the sulfur S_8_ ring opens, resulting in the formation of high-order polysulfides Li_2_S_*x*_ (6 < *x* ≤ 8). As the cell continues to discharge and lithium cations are incorporated, lower-order polysulfides are formed – Li_2_S_*x*_ (2 < *x* ≤ 6).^14,15^ Because of the complexity of this reaction, lithium–sulfur batteries face several challenges that limit their practical use. One of the main problems is the “shuttle effect”, where lithium polysulfides dissolve in the electrolyte and move between the cathode and anode.^16,17^ This leads to a gradual loss of active material,^18^ reduced Coulombic efficiency,^19^ and faster capacity fading.^20^ Additionally, the shuttle effect can cause self-discharge and damage the lithium metal anode, further shortening the battery’s lifespan.^21^ These issues, along with the poor conductivity of sulfur^22^ and significant volume changes during cycling,^23^ make it difficult to achieve long-term stability and performance in lithium–sulfur batteries.

To solve these problems, many pathways have already been proposed. In order to improve the capacity of lithium–sulfur batteries and reduce the shuttle effect, several modifications can be made. One of the most examined approaches is the use of advanced cathode host materials, such as porous carbon frameworks, metal-organic frameworks (MOFs), carbon nanotubes, or conductive polymers.^24–29^ These materials provide a large surface area and high conductivity, which accommodate the volume expansion of sulfur while also confining the soluble polysulfide intermediates that tend to diffuse into the electrolyte because of Lewis acid–base interactions.^30^

Another promising strategy involves the incorporation of polar or chemically functionalized materials, such as metal oxides (e.g., TiO_2_, MnO_2_),^31,32^ sulfides,^33,34^ or nitrogen-doped graphene.^35,36^ These polar surfaces have strong chemical interactions with lithium polysulfides, effectively anchoring them and reducing their mobility within the cell.^37^

In addition, physical barriers, such as modified separators or interlayers coated with conductive and polysulfide-trapping materials, can be inserted between the electrodes. These interlayers act as additional protective barriers, preventing polysulfide migration while maintaining efficient ion transport.^38,39^

Also, carbon conductive additives play a crucial role in enhancing the electrochemical performance of lithium–sulfur batteries. Due to the inherently low electrical conductivity^40^ of sulfur and its discharge products, the inclusion of conductive carbon materials—such as carbon black,^41,42^ nanofibers,^43^ carbon nanotubes,^27^ porous hollow carbon spheres^44^ or graphene^45^—is essential to facilitate electron transport within the cathode. These additives create a conductive network that improves the utilization of active material, enables more uniform reaction kinetics, and also helps physically trap polysulfides.

Electrolyte engineering also plays a crucial role in enhancing battery performance. The use of high-concentration electrolytes or solid-state electrolytes can suppress polysulfide dissolution and limit side reactions with the lithium anode.^46^ Additives such as lithium nitrate (LiNO_3_) are often introduced to form a stable solid electrolyte interphase (SEI) on the lithium surface, which further protects the anode from unwanted reactions.^47^ As the electrolyte consists of solvents, salt, and additives, the variety of modifications to the solution is wide. Especially, changes in the salt seem to be a promising path in order to replace the most popular one used in lithium–sulfur technology—lithium bis­(trifluoromethanesulfonyl)­imide (LiTFSI). LiTFSI salt in the electrolyte solution neither reduces the shuttle effect nor prevents polysulfide dissolution and their composition.^48^ Also, it contains a total of six fluorine atoms per molecule, which makes it not particularly eco-friendly.

In this research, we are focusing on two aspects of modifications for lithium–sulfur batteries. The first aspect is cathode engineering, whose goal is to obtain simple, easy-to-prepare and low-cost model cathodes that enable the simulation of cell cycling with different sulfur availability. We aim to find the dependence and impact of all the components of the electrode on the following parameters: sulfur loading, electronic conductivity, electrolyte-to-sulfur ratio (E/S), the impact of the binder, and general electrochemical performance. To achieve this, we compare: 1) three different carbon blacks: Super P, Ketjen Black EC-600-JD, and Vulcan XC-72; 2) sodium carboxymethyl cellulose (NaCMC) polymers (Mw = 250 000 and 700 000); 3) NaCMC concentration in water solution; and 4) the mass percentage of sulfur in the electrode mass. The aim of this part of the work is to build simple model cathodes (easy to prepare, cheap, and universal) that can be widely used with different electrolytes. The second aspect of the research is the electrolyte. Many studies have been conducted in recent works to improve the performance of this battery component. The possibilities of applying weakly solvating electrolytes (WSEs)^49,50^ in order to reduce anode degradation, improve sluggish cathode kinetics, or introduce ammonium cations to limit the reaction of polysulfides with the anode^51^ are possible ways to improve and better understand electrolyte chemistry in the battery. We decided to implement a variety of new types of salts in order to improve battery performance—Hückel anion-based salts that we have been developing for almost 20 years with our team.^52^ We compare the performance of lithium 4,5-dicyano-2-(n-heptafluoropropyl)­imidazolide (LiHDI), lithium 4,5-dicyano-2-(pentafluoroethyl)­imidazolide (LiPDI), lithium 4,5-dicyano-2-(trifluoromethyl)­imidazolide (LiTDI), and lithium 2,4,5-tricyanoimidazolide (LiTIM). The last of the salts listed is fluorine-free, making it a good fit for the challenges of today’s battery engineering. Altogether, these modifications aim to significantly improve the cyclability, Coulombic efficiency, and overall energy density of lithium–sulfur batteries, bringing them closer to practical, commercial applications in everyday devices. This work is a broader continuation of our previous studies on Hückel anion-based salts in Li–S systems, where we have shown and explained why such anion-based electrolytes, with tailored salt ratios, hold significant promise for advancing future Li–S battery technologies by presenting their advantages at C/10 cycling.^46,53,54^

Overall, this study provides a coherent and systematic assessment of cathode formulation and electrolyte salt chemistry in Li–S batteries, offering experimentally grounded insights into their combined impact on cell performance. The presented results support the rational design of Li–S systems employing alternative lithium salts and optimized cathode architectures, contributing to the development of more stable and efficient next-generation energy storage technologies.

## 2 Experimental and Methods

### 2.1 Electrode Fabrication

Electrodes were made of sulfur (Sigma-Aldrich, 99.998% trace metal basis) and differed in the type of carbon black and binder used. To prepare the electrodes, different amounts of sulfur and carbon blacks were mixed in a mortar. Next, the binder solution was added, and the samples were mixed on a magnetic stirrer to obtain a homogeneous slurry. All of the electrode suspensions were coated on 20 μm aluminum foil (Hohsen) using the Doctor Blade technique. The thickness of all the layers was set to 250 μm. After coating, the electrodes were dried at a temperature of 60 °C in a vacuum dryer for 24 h. As carbon black additives, Super P (Imerys), Vulcan XC-72 (Timcal), and Ketjen Black EC-600JD were used. As binders, two different sodium carboxymethyl cellulose (NaCMC) types were used, differing in molecular weight (average Mw = 250 000, Ds = 1.2 (Sigma-Aldrich), and average Mw = 700 000 (Sigma-Aldrich)). The solutions also differed in the concentration of NaCMC dissolved in water. The compositions of the electrodes are shown in Table 1.

**Table 1:** Electrode Compositions Considered in the Research

Sulfur percentage (weight)	Carbon black type	Binder type
60%	Super P	average Mw = 250 000, Ds = 1.2
		average Mw = 700 000
	Ketjen Black EC-600JD	average Mw = 250 000, Ds = 1.2
		average Mw = 700 000
	Vulcan	average Mw = 250 000, Ds = 1.2
		average Mw = 700 000
70%	Super P	average Mw = 250 000, Ds = 1.2
		average Mw = 700 000
	Ketjen Black EC-600JD	average Mw = 250 000, Ds = 1.2
		average Mw = 700 000
	Vulcan	average Mw = 250 000, Ds = 1.2
		average Mw = 700 000

### 2.2 Electrolyte Preparation

In the research, five different electrolytes were used: 1 M LiTFSI (Sigma-Aldrich) in DOL/DME 1:1 w/w (Sigma-Aldrich) with the additive of 1% LiNO_3_ (Sigma-Aldrich), 1 M LiTDI in DOL/DME 1:1 w/w with the additive of 2% LiNO_3_ (Sigma-Aldrich), 1 M LiPDI in DOL/DME 1:1 w/w with the additive of 2% LiNO_3_ (Sigma-Aldrich), 1 M LiHDI in DOL/DME 1:1w/w with the additive of 2% LiNO_3_ (Sigma-Aldrich), and 1 M LiTIM in DOL/DME 1:1 w/w with the additive of 2% LiNO_3_ (Sigma-Aldrich). The structures are shown in Figure 1. The LiTDI, LiPDI, LiHDI, and LiTIM salts were synthesized at Warsaw University of Technology (using a protocol described by Niedzicki et al. and Żukowska et al.).^55,56^

**Figure 1:**
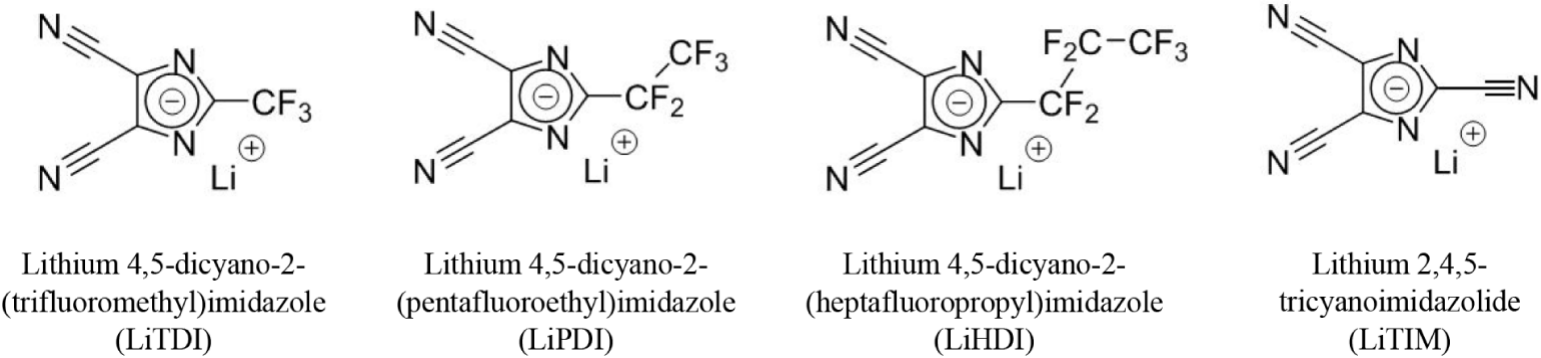
Hückel anion-based salt structures used in the current work.

### 2.3 Cell Preparation

Cells were prepared as half-cell coin cell-type batteries (CR2032) in an argon-filled MBraun glovebox chamber under an atmosphere of <0.1 ppm O_2_ and <0.1 ppm of H_2_O. To prepare the cells, circle-shaped electrodes were cut from previously prepared layers. The diameter of the electrodes was set at 11 mm. After cutting, all the electrodes were pressed under 6 t cm^2^ at manual press. Half-cells were assembled using lithium (Sigma-Aldrich), a size-fitted Celgard separator placed between the positive and negative electrodes, and a proper electrolyte solution prepared beforehand. The amount of electrolyte added to each cell was fixed at 50 μL.

### 2.4 Electrochemical Tests

Cell electrochemical characterization was carried out using a multichannel VMP3 BioLogic potentiostat with the GCPL method. The cycling potential range was set to 1.7 V–2.7 V vs metallic lithium. Initially, each cell was formed one time at the current C/100. Then, for the rate test, a series of cycles were carried out, differing in the currents: C/20, C/10, C/5, C/2, and C/5. For each current, 3 repetitions of cycling were performed. For the long cycling test, after the formation cycle with C/100, 100 repetitions of charge and discharge cycles were carried out at the current C/5.

### 2.5 Electrode Resistance Tests

The resistance of the sulfur electrodes was measured using the four-point probe method. In the tests, four tungsten blades were used. The range of currents was set from 10^–6^ A to 10^–3^ A. The sampling time was 30 s, and for each current value, 10 repetitions of the experiment were conducted. Then, the average value of the voltage was calculated in order to obtain the resistance values.

### 2.6 SEM and EDS Electrode Examination

For elemental mapping analyses, an SEM/FIB system (Helios 600 NanoLab Dual Beam FEI) equipped with the QUANTAX FlatQUAD (Bruker) Energy Dispersive Spectroscopy (EDS) detector was used. Elemental mapping analyses were performed on the surfaces and cross-sections of the examined samples. Cross-sections were fabricated by site-specific material removal using a focused gallium ion beam (FIB) milling process in two steps: coarse milling and cleaning milling. The FIB processes leading to the fabrication of cross-sections were carried out using an ion beam with an energy of 30 kV and an ion-beam current adjusted within the range from nanoamperes to picoamperes. All samples were maintained at a temperature of −40 °C during SEM observation, FIB preparation, and EDS analysis. Cooling of the specimens was required to prevent sulfur sublimation from the samples under high-vacuum conditions during SEM/FIB experiments.

### 2.7 Raman Spectroscopy

The samples for the Raman spectroscopy measurements were 0.5 cm^3^ and were sealed in 2 cm^3^ glass vials. The spectra were collected on a Nicolet Almega Raman dispersive spectrometer using a diode laser with an excitation wavelength of 532 nm and a spectral resolution of 2 cm^–1^. The background subtraction of spectra was done using the OMNIC software (OMNIC, Thermo Scientific) and the spectra were normalized in 900 to 800 cm^–1^ spectral range.

The number of coordinated solvent molecules (N) in the primary solvation shell of Li was calculated based on the relationship between (i) the Raman intensities of the peaks corresponding to bound (*I_b_*) and free (*I_f_*) solvent molecules—assuming similar Raman scattering factors—and (ii) the concentrations of the Li salt (*c_Li_*) and the solvent (*c_solvent_*). The relationship is expressed as follows:

1 $$\frac{I_{f}}{I_{b}+I_{f}}=N\frac{c_{Li}}{c_{solvent}}$$

## 3 Results and Discussion

### 3.1 Optimization of the Model Cathodes

Obtaining model lithium–sulfur cathodes requires an optimization process that covers the variety of components involved in electrode fabrication, such as diverse conductive carbons, binders, and sulfur loadings, each of which can profoundly influence the final electrochemical and physicochemical properties. By comprehensive evaluation of these variables, optimal compositions can be identified to maximize capacity, rate capability, and cycling stability. Such optimization results, including visual homogeneity, performance tests, the effect of the carbon-to-sulfur ratio, the impact of carbon porosity, and sulfur utilization parameters, are presented in the SI (Sections 1.1, 1.2, 1.3, and 1.4).

#### 3.1.1 Effect of Sulfur Loading

Sulfur loading directly affects the capacity and practical energy density of lithium–sulfur batteries. Higher sulfur loading can increase the areal capacity, making the battery more suitable for applications. However, excessive loading often leads to poor electrolyte infiltration and limits the reaction kinetics, which can result in rapid capacity fading. Balancing sulfur loading with proper electrode design is essential to maintain structural integrity, ensure efficient ion and electron transport, and achieve long-term cycling stability. Figure 2a presents the changes in the milligrams of sulfur per cm^2^ of the electrode depending on the percentage of sulfur in the electrode mass and the NaCMC average polymer molecular weight. For the electrode containing 60% of sulfur, the amount of active material is similar (around 1.7 mg S/cm^2^). For the electrode with 70% of sulfur the differences are more visible. More favorable in terms of sulfur loading seems to be NaCMC of average Mw = 250 000 (above 2.5 mg S/cm^2^) compared to NaCMC of average Mw = 700 000 (about 1.7 mg S/cm^2^). Figure 2b the electrochemical performance and the capacities of the electrodes were compared. Both 70% sulfur electrodes reached similar capacities. For the electrode with 70% S/20% C/10% NaCMC 700 000, the average from three repetitions was: 567 mAh g^–1^ for C/20, 431 mAh g^–1^ for C/10, 256 mAh g^–1^ for C/5, and 139 mAh g^–1^ for C/2. For the electrode with 70% S/20% C/10% NaCMC 250 000, the average from three repetitions: 511 mAh g^–1^ for C/20, 454 mAh g^–1^ for C/10, 238 mAh g^–1^ for C/5, and 125 mAh g^–1^ for C/2. The differences are more visible for the electrode with 60% sulfur, which exhibited slightly lower capacities at C/10 and C/5 rates, delivering 290 and 175 mAh g^–1^, respectively.

**Figure 2:**
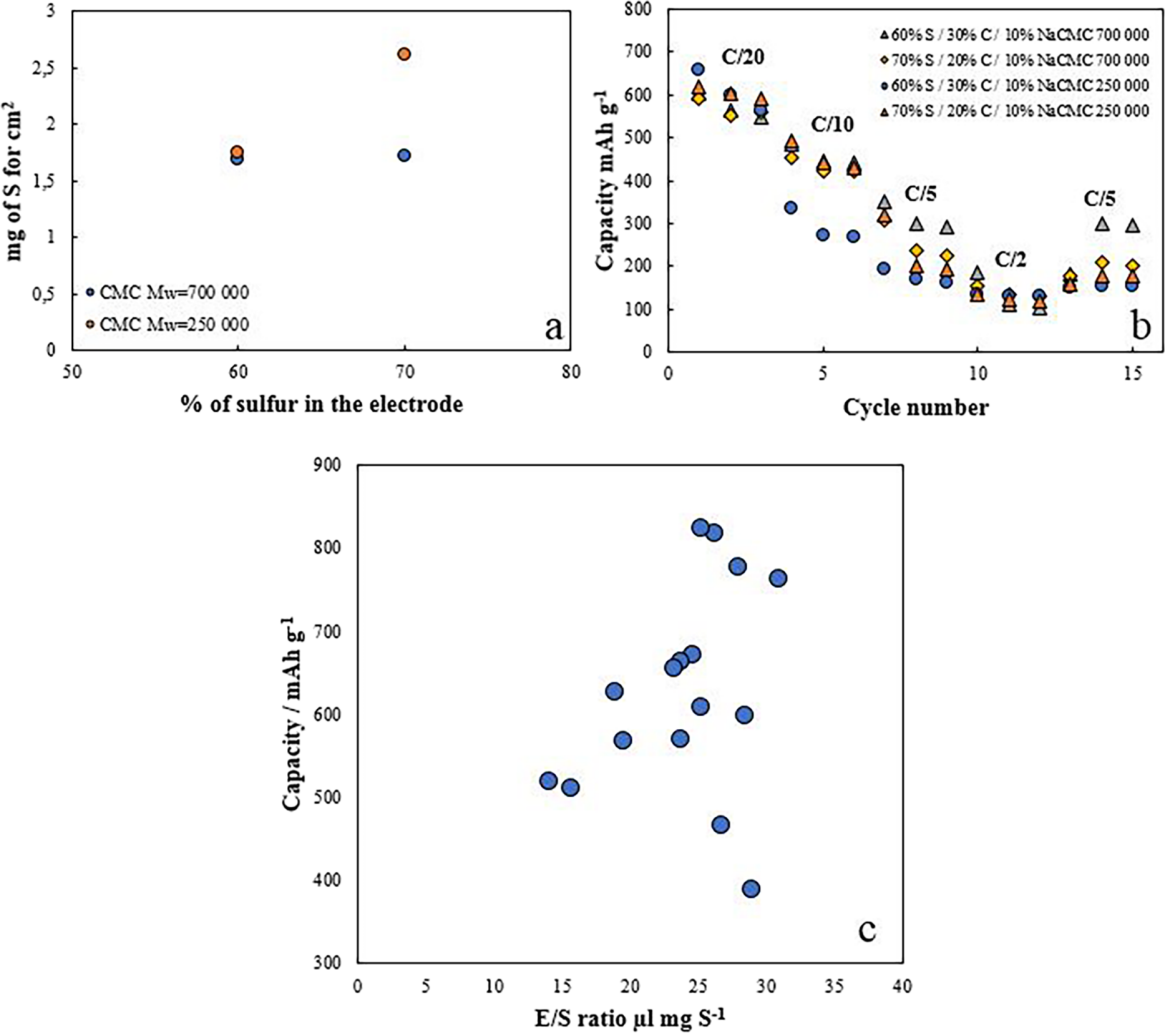
(a) Electrode sulfur loading (mg/cm²) (b) electrochemical performance of the electrodes vs sulfur loading, and (c) E/S ratio performance comparison for the current C/20.

#### 3.1.2 E/S Ratio

The electrolyte-to-sulfur (E/S) ratio is a critical parameter in the design and performance of Li–S batteries. It refers to the volume or weight of electrolyte used per unit mass of sulfur in the cathode. A lower E/S ratio is desirable for achieving high energy density, as excessive electrolyte reduces the overall specific energy of the cell. However, reducing the E/S ratio too much can compromise ionic conductivity, hinder sulfur redox reactions, and accelerate capacity fading due to insufficient wetting and polysulfide solubility. Optimizing the E/S ratio is essential for balancing energy density, cycle life, and the practical viability of Li–S batteries. Figure 2c illustrates the relationship between specific capacity and the electrolyte-to-sulfur (E/S) ratio. The highest specific capacity, approximately 800 mAh g^–1^, was achieved at an E/S ratio of 20–25 μL/mg S, indicating this as the optimal value within the tested range. In the current report, the investigated E/S ratio is in the range of 14–31 μL/mg S. However, when the E/S ratio exceeded 25 μL/mg S, the specific capacity began to decline, suggesting that excessive electrolyte negatively impacts cell performance.

#### 3.1.3 Electrode Layer Electronic Conductivity Test

Electronic conductivity plays a vital role in the performance of Li–S batteries, particularly due to the inherently low conductivity of elemental sulfur. This poor conductivity hinders efficient electron transport during electrochemical reactions, limiting the utilization of active material and reducing the battery’s rate capability and overall energy efficiency. To conclude the optimization analysis in Section 3.1, electronic conductivity tests were conducted on the electrodes. As shown in Table 2, Vulcan exhibited the lowest average electronic resistance at 31.03 Ω, indicating the highest electronic conductivity among the tested carbon blacks. Super P showed a higher resistance of 56.63 Ω, while Ketjen Black had the highest resistance at 60.33 Ω. These results may seem counterintuitive when compared to the electrochemical performance discussed earlier. Such relatively small discrepancies suggest that the percolation threshold has been effectively reached in all studied cathode formulations, ensuring that the conductive network is sufficiently developed to support electron transfer without becoming the primary limiter for the redox kinetics. Consequently, the observed results imply that under these specific experimental conditions, the battery’s performance is not limited by electronic conductivity but rather by ionic transport and the availability of active sites. In Li–S systems, the electrochemical behavior is heavily dictated by the management of soluble lithium polysulfides. The high porosity of Ketjen Black (shown in Table 2) acts as an effective physical and adsorptive barrier that anchors polysulfides within the cathode structure, significantly reducing their dissolution into the electrolyte and mitigating the shuttle effect. This structural advantage more than compensates for the marginal increase in electronic resistance.

**Table 2:** Resistance Values of the Electrodes Containing 60% of Sulfur and Carbon Blacks Used in the Research Depending on the Applied Current

	Vulcan XC-72				Super P				Ketjen Black EC-600JD			
Specific surface area [m^2^ g^–1^]	**250** ^57^				**64** ^58^				**1400** ^59^			
Current [A]	10^–6^	10^–5^	10^–4^	10^–3^	10^–6^	10^–5^	10^–4^	10^–3^	10^–6^	10^–5^	10^–4^	10^–3^
Average [mV]	0.030	0.330	3.399	34.210	0.051	0.575	5.880	59.181	0.062	0.541	6.304	62.367
Resistance [Ω]	29.90	33.02	33.99	34.21	51.10	57.45	58.80	59.18	61.80	54.12	63.04	62.37
Average resistance [Ω]	**31.03**				**56.63**				**60.33**			

On the basis of the results presented in Section 3.1, the best-performing cathodes were selected for further investigation. The primary criteria for selection were a high specific capacity and consistent performance across multiple cycles. Three electrodes were identified as the most promising: one containing 60% sulfur, Super P carbon black, a NaCMC binder with a molecular weight of 700 000, and 2.0% binder concentration; another with 60% sulfur, Ketjen Black, the same binder specifications; and a third with 60% sulfur, Vulcan XC-72 carbon black, and a slightly lower binder concentration of 1.5%. These electrodes demonstrated the most stable and repeatable electrochemical performance among all of the samples tested during the optimization phase. The deliberate selection of electrodes incorporating three different carbon blacks allows for a comparative evaluation of the impact of the carbon type on performance. Among them, the electrode with Ketjen Black EC-600-JD achieved the highest capacities, while the Super P-based electrode showed the lowest. In the following section, only these three optimized electrodes will be used to evaluate the performance of new electrolyte solutions.

### 3.2 New Electrolytes Tests

The optimization shown in Section 3.1 allowed us to choose the three best-performing electrodes. These electrodes were subsequently tested with the electrolytes listed in Section 2.2. The tests were performed in the same way as that presented before. Each battery was prepared as a half-cell with metallic lithium as the negative electrode. The GCPL method with different currents was the same as in the optimization protocol; the battery was charged and discharged according to the sequence: C/20, C/10, C/5, C/2, and C/5. The results and a comparison of the performance of the electrolytes are presented in the following sections.

#### 3.2.1 Different C-Rates Capacity Tests

Figures 3, 4, and 5 present a comparative analysis of the electrochemical performance of three electrodes using different carbon black cathode materials—Super P, Vulcan XC-72, and Ketjen Black EC-600JD—combined with five different electrolyte salts: the conventional LiTFSI, and four alternative lithium salts (LiTDI, LiPDI, LiHDI, and LiTIM). The influence of the carbon structure and electrolyte composition on capacity retention and overall performance is clearly visible in the results.

**Figure 3:**
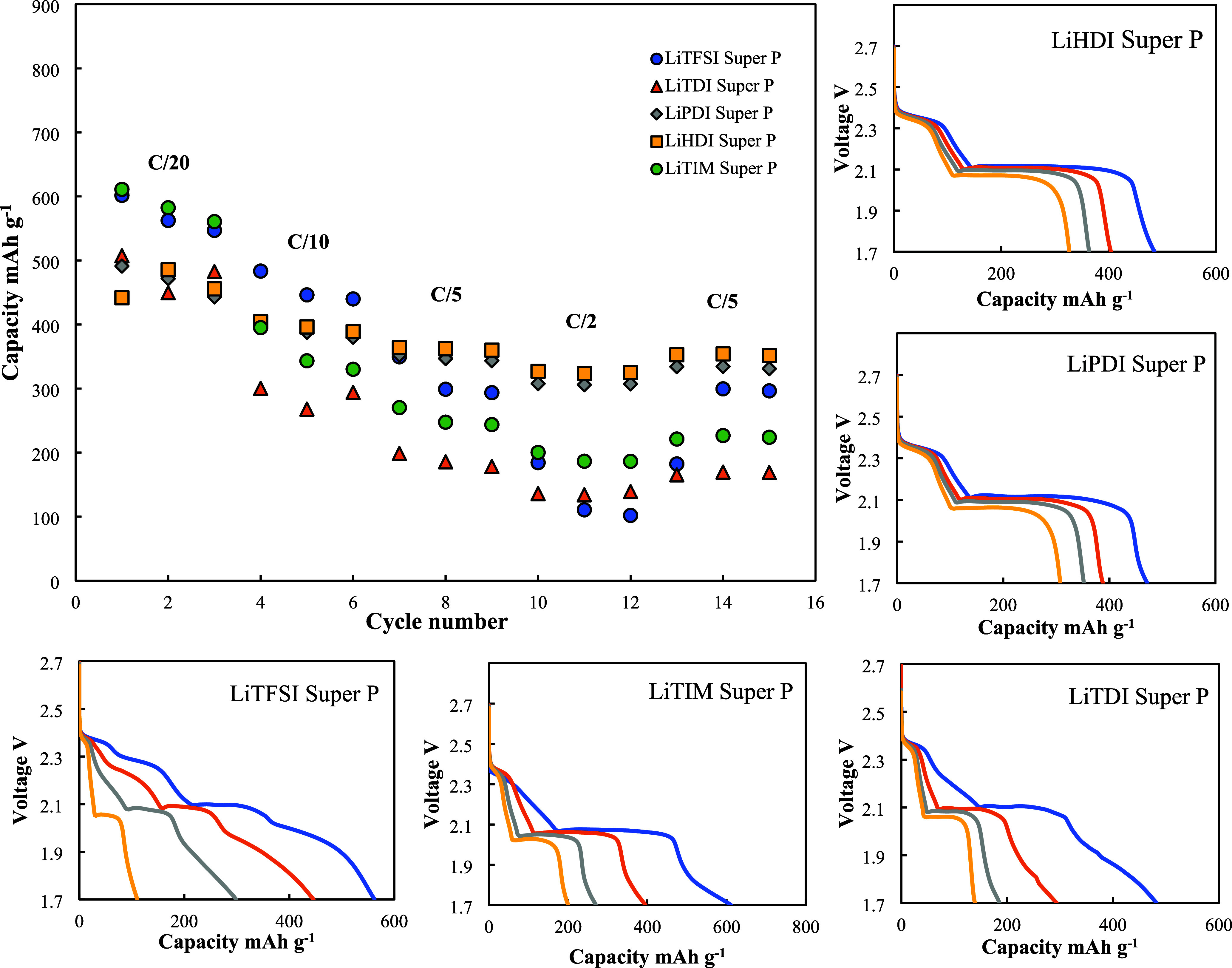
High-rate tests of the cells with Super P carbon black, along with discharge curves, are presented for each electrolyte (on the discharge curve plots, the color meanings are as follows : blue—C/20, orange—C/10, gray—C/5 and yellow—C/2).

**Figure 4:**
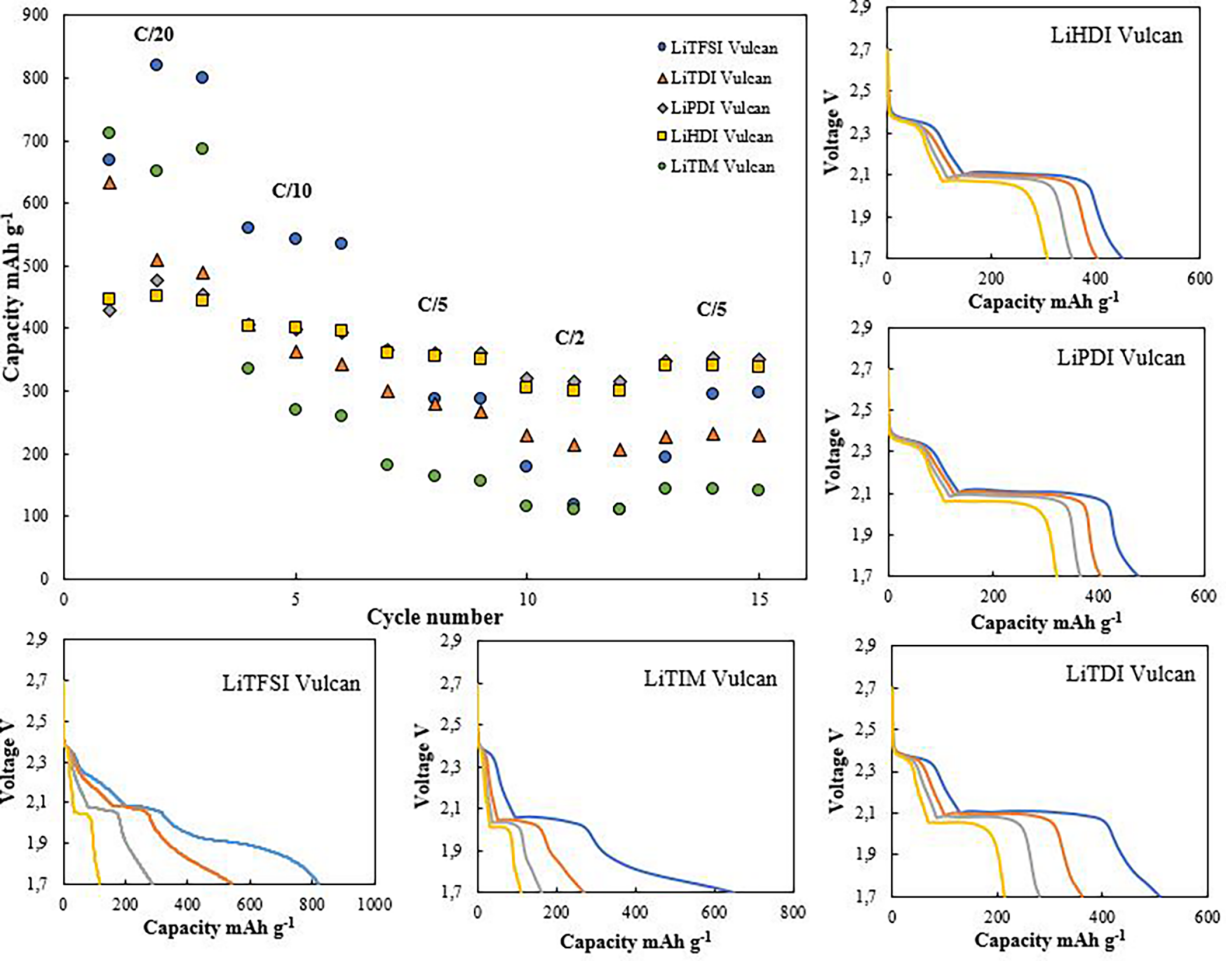
High-rate tests of the cells with Vulcan carbon black, along with discharge curves, are presented for each electrolyte (on the discharge curve plots, the color meanings are as follows: blue—C/20, orange—C/10, gray—C/5 and yellow—C/2).

**Figure 5:**
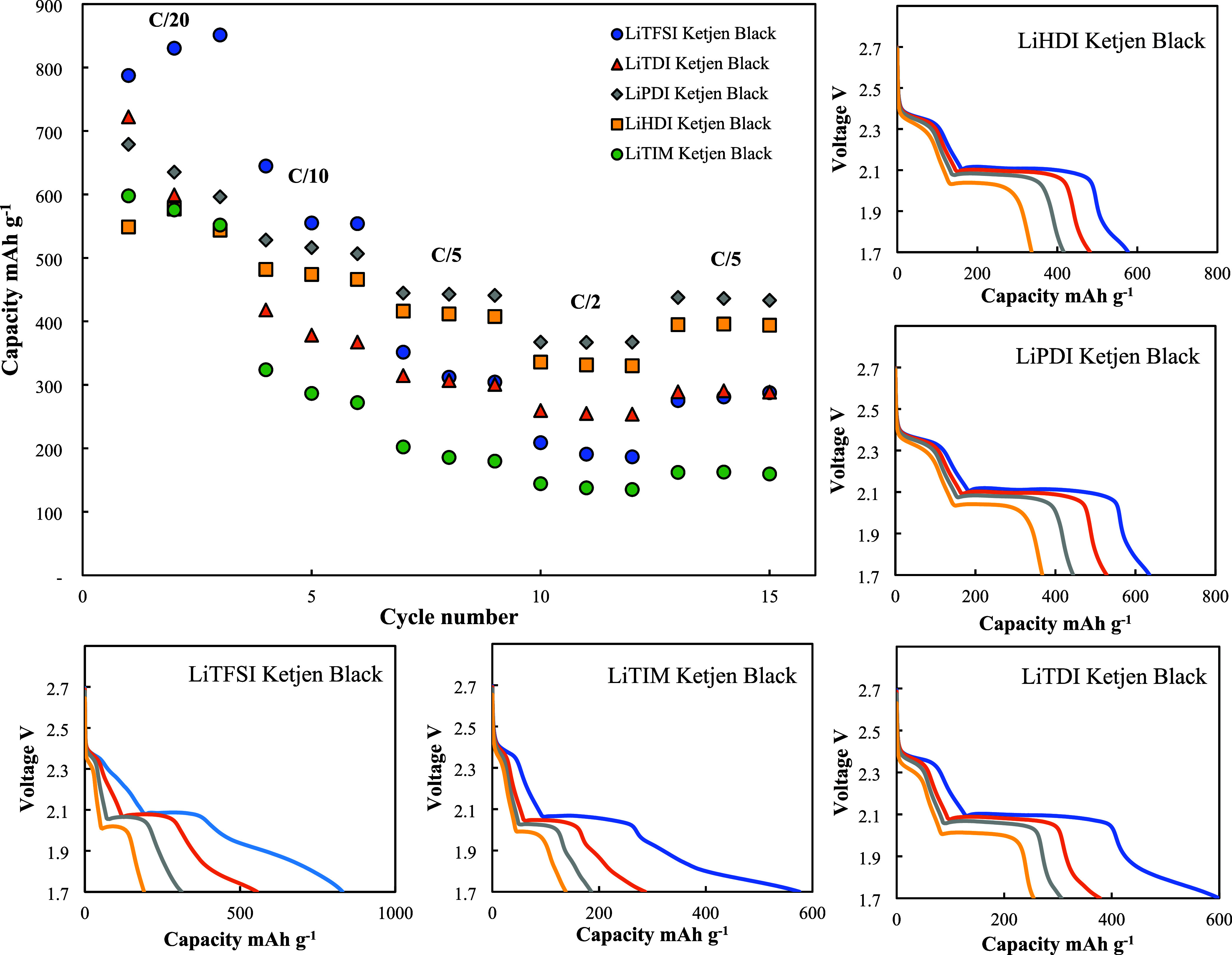
High-rate tests of the cells with Ketjen Black carbon black, along with discharge curves, are presented for each electrolyte (on the discharge curve plots, the color meanings are as follows : blue—C/20, orange—C/10, gray—C/5 and yellow—C/2).

Across all three carbon blacks, LiTFSI—the industry-standard salt —delivers the highest initial capacities at the rate C/20, reaching the range of 600 to 850 mAh g^–1^, regardless of the carbon black used. However, this performance declines significantly at higher rates (C/2), demonstrating a limited ability for faster charging. These results suggest that LiTFSI may not sufficiently mitigate polysulfide dissolution or maintain stable interfacial properties, even when paired with high-surface-area conductive additives.

In contrast, while the initial capacities of the salts LiHDI and LiPDI are lower (440 to 680 mAh g^–1^), they perform excellently at higher rates, showing at the same time stable capacity retention over time and the highest capacities for rates C/5 and C/2 among all tested solutions. This trend continues with all types of carbons. It indicates a trade-off between initial performance and long-term cycling stability when selecting electrolytes for Li–S batteries.

For LiTDI paired with Super P, the performance at the current rate of C/20 is similar to that of LiPDI and LiHDI, but for faster rates (C/10 and C/5), the specific capacity decreases and is the lowest among the tested solutions. For the electrodes with Vulcan XC-72 and Ketjen Black EC-600JD, the performance at C/5 and C/2 is much better. Ketjen Black’s superior performance under these rates reinforces the benefit of high-surface-area conductive networks in maintaining active sulfur utilization. The electrodes are reaching capacities comparable to or slightly higher than those of the LiTFSI electrolyte.

Notably, LiTIM, an entirely fluorine-free salt, also offers respectable performance with all three carbon blacks. Although its initial capacities are modest compared to those of LiTFSI, it demonstrates competitive stability, particularly when combined with Vulcan and Ketjen Black. This indicates that, while LiTIM on its own may not offer high electrochemical activity, its performance can be significantly enhanced through the appropriate selection of the conductive additive. The environmental benefits of its fluorine-free structure further support the continued development of this system, provided that its limitations in electrochemical stability can be mitigated.

The influence of the carbon black type was also evident throughout the study. Super P, a widely used conductive carbon with a relatively low surface area, consistently resulted in the lowest capacities and the most pronounced capacity fade, regardless of the electrolyte salt. Vulcan showed moderate improvement, enabling slightly better retention and higher capacities. Ketjen Black, with its high surface area and superior conductive network, significantly improved cell performance across all salts, particularly for LiPDI, LiHDI, and LiTIM. This underscores the critical role of conductive additive morphology and porosity in facilitating sulfur utilization and trapping soluble polysulfide intermediates.

In summary, the study highlights the complex interplay between the cathode material and electrolyte composition in Li–S battery performance. While LiTFSI remains the benchmark for high initial capacities, newer salts like LiHDI and LiPDI offer better long-term stability and higher capacities for the rates of C/5 and C/2, especially when combined with high-surface-area carbons such as Ketjen Black. The fluorine-free LiTIM, although slightly lower in capacity, stands out as a sustainable alternative. These findings underscore the need to optimize both electrolyte formulation and cathode design to balance energy density, cycle life, and environmental impact.

Figures 3, 4, and 5 also present the discharge voltage profiles of the investigated cells. Although minor differences are observed, similar overall trends are evident for all three carbon blacks when LiTDI, LiPDI, and LiHDI electrolytes are used.

For cells containing LiHDI and LiPDI, two well-defined discharge plateaus are observed. The first plateau, occurring in the voltage range of 2.3–2.4 V, corresponds to the formation of long-chain lithium polysulfides (Li_2_S_8_, Li_2_S_6_, and Li_2_S_4_). The second plateau, located at 2.0–2.1 V, is associated with the conversion of these species into short-chain polysulfides (Li_2_S_2_ and Li_2_S).

In the case of the LiTDI electrolyte, the first plateau is noticeably shorter compared to LiPDI and LiHDI, indicating a reduced formation of long-chain polysulfides. For electrodes based on Super P carbon black, the discharge curve obtained at a C/20 rate exhibits a smoother voltage decay in the transition region between the first and second plateaus compared to those measured at higher C-rates (C/10, C/5, and C/2), as well as relative to Ketjen Black and Vulcan carbon blacks. This behavior suggests an increased formation of intermediate polysulfides, particularly Li_2_S_6_/Li_2_S_4_.

For the LiTIM electrolyte, the first discharge plateau is significantly shorter than that observed for LiTDI and is even less pronounced compared with LiPDI and LiHDI, further indicating limited formation of long-chain polysulfides. Also, a similar trend to that observed for LiTDI is seen for Super P carbon black: at a C/20 rate, the transition region between the two plateaus is smoother than at higher current densities and compared to those of Ketjen Black and Vulcan carbons, implying enhanced generation of Li_2_S_6_/Li_2_S_4_ polysulfides.

When LiTFSI is employed as the electrolyte salt, the most pronounced differences among the carbon black samples are observed. For Super P, the discharge curves at C/20 and C/10 exhibit multiple distinct regions within the first plateau, indicating a sequential formation process in which Li_2_S_8_ forms initially, followed by Li_2_S_6_/Li_2_S_4_. A similar but less pronounced behavior is observed for Vulcan carbon black, with shorter plateau regions. In contrast, this tendency is only weakly evident for Ketjen black and is almost absent in the C/10 discharge profile.

Across all discharge curves, an overall downward shift of the plateau voltages is observed with increasing current rates. This effect is attributed to an increased level of polarization associated with higher current densities.

#### 3.2.2 Long-Term Cycling

To test the electrolyte solutions and carbon blacks further, long-term cycling was performed. The selected electrodes were the same as for Section 3.2.2. Figure 6 represents the results for the electrodes with Super P, Figure 7 shows the results with Vulcan XC-72, and Figure 8 illustrates the results for Ketjen Black EC-600JD. Tests were performed at a C/5.

**Figure 6:**
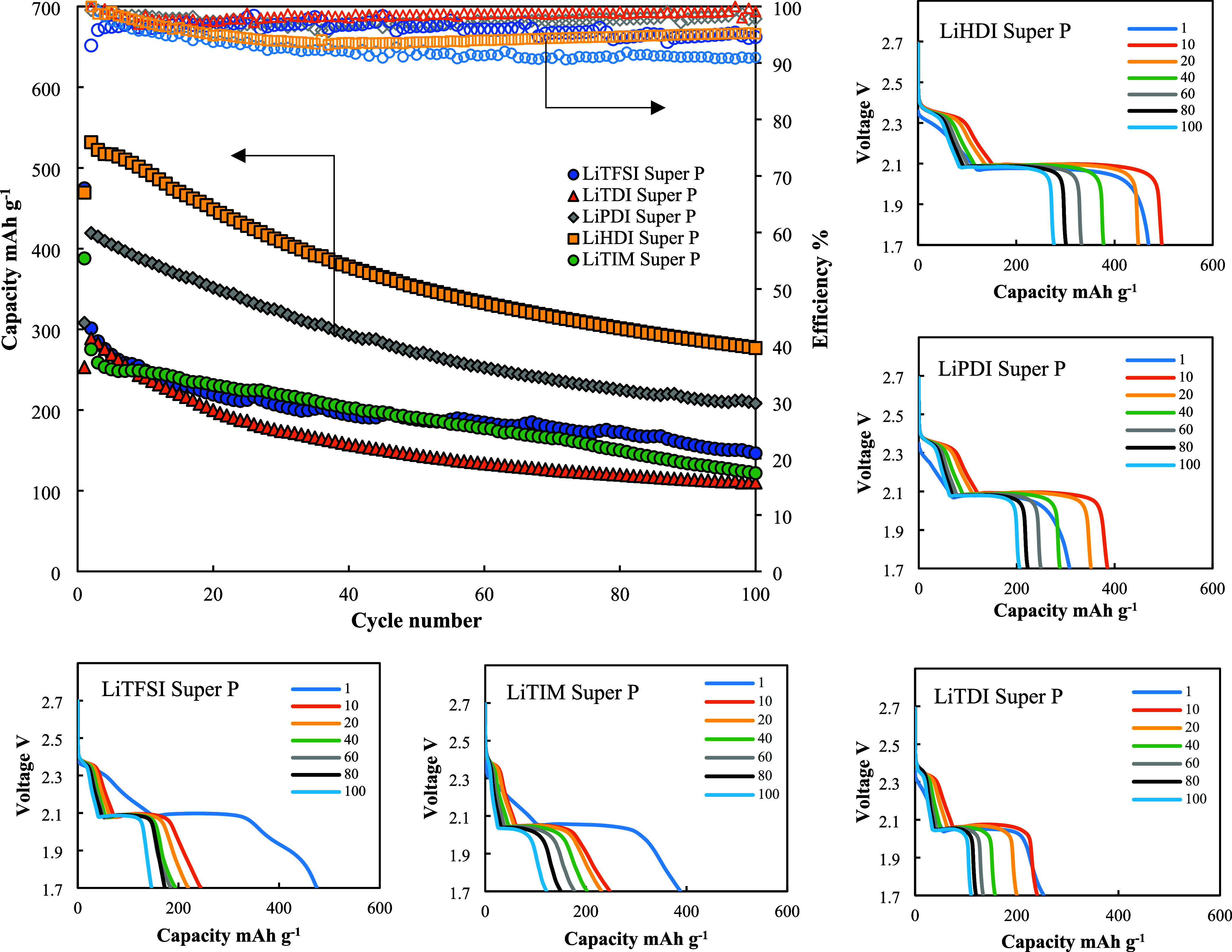
Long cycling tests of the cells with Super P carbon black, along with discharge curves, are presented for each electrolyte.

**Figure 7:**
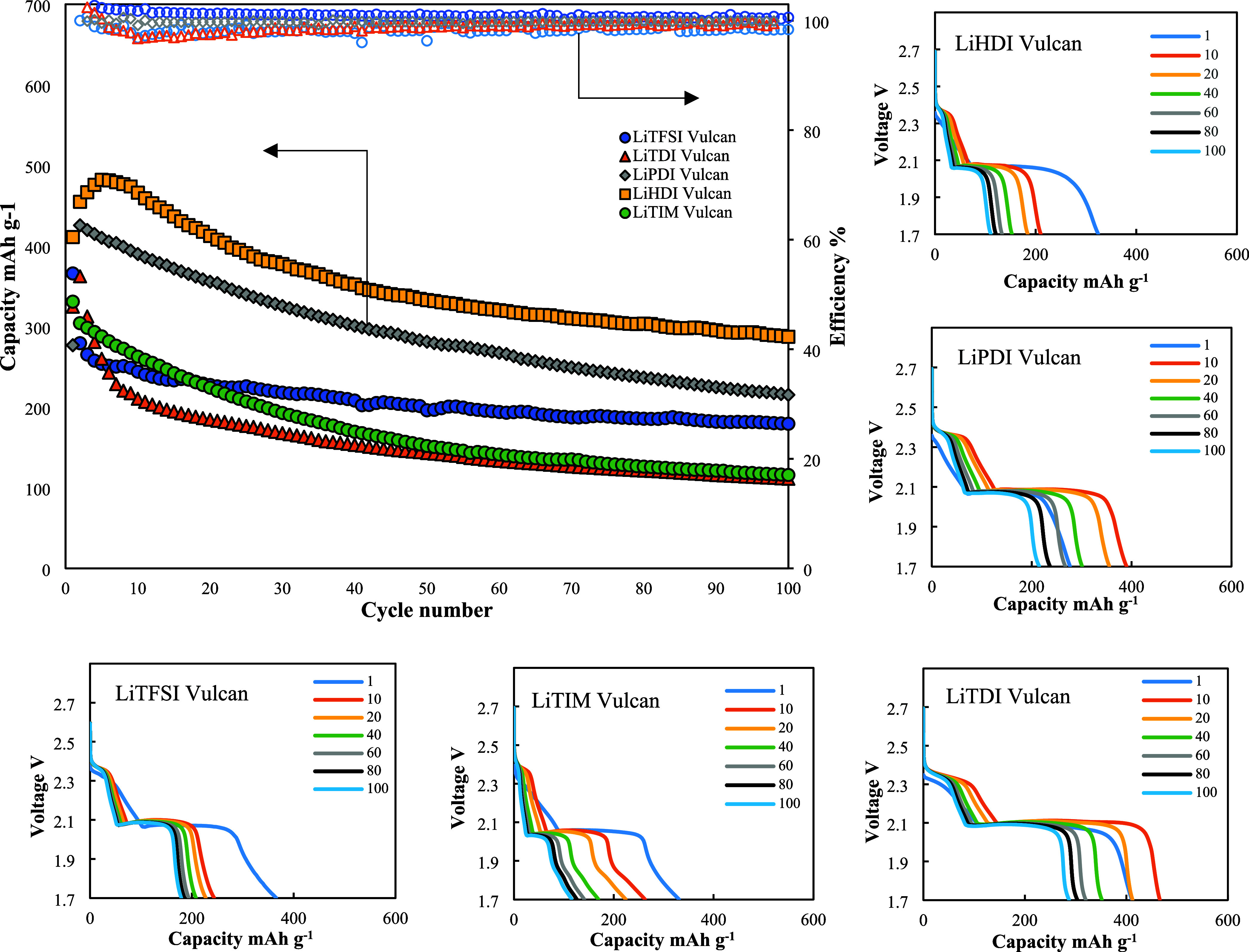
Long cycling tests of the cells with Vulcan carbon black, along with discharge curves, are presented for each electrolyte.

**Figure 8:**
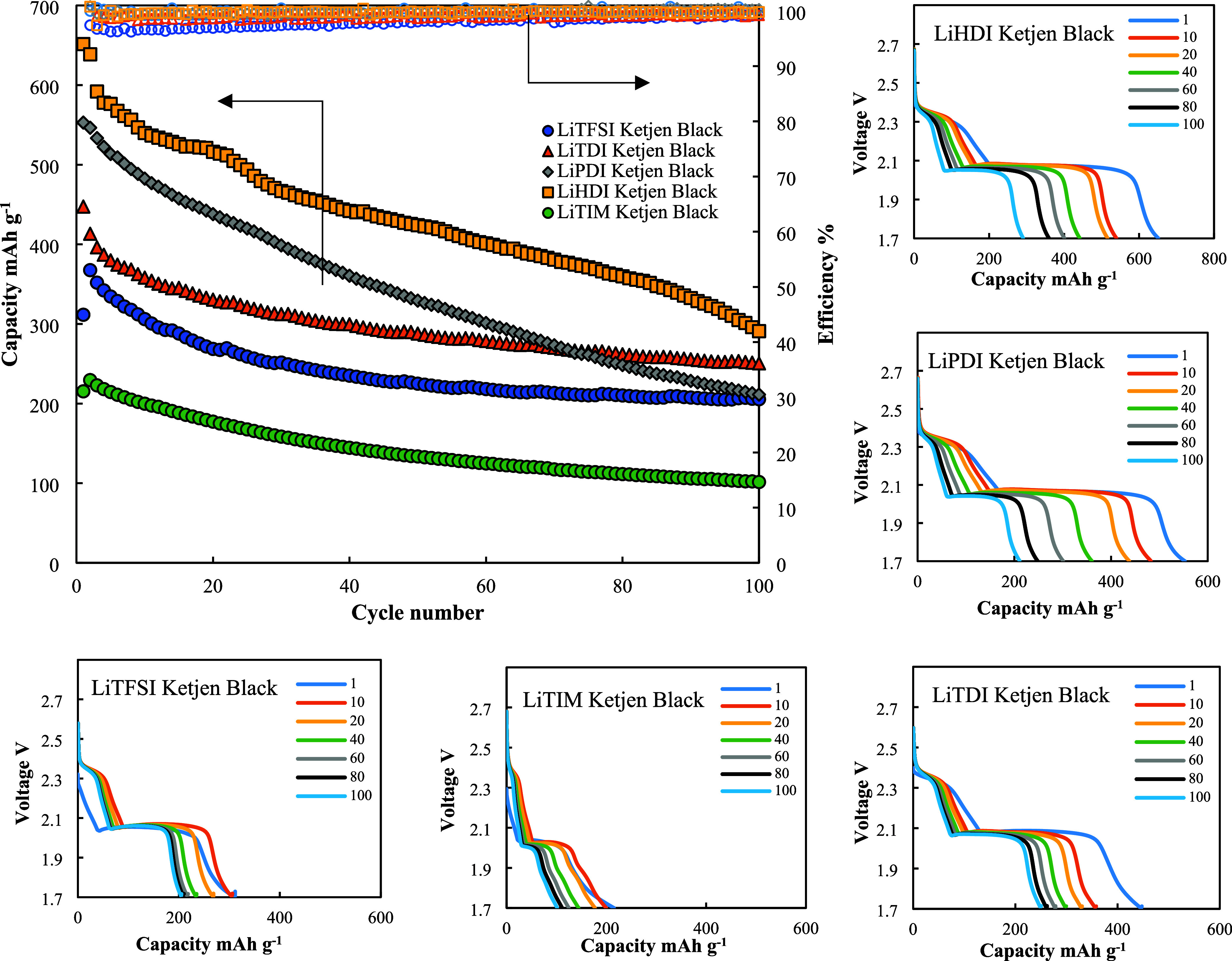
Long cycling tests of the cells with Ketjen Black carbon black, along with discharge curves, are presented for each electrolyte.

For the Super P electrodes (Figure 6) the best-performing electrolyte solution was the one containing LiHDI. Starting with an initial capacity of 530 mAh g^–1^, the capacity fade was rather significant (48% capacity fade comparing to first cycle). LiPDI solution performed slightly worse, starting with 420 mAh g^–1^, the capacity fade was 50%. As for the LiTFSI and LiTIM, the performance was quite similar (starting in c.a 310 mAh g^–1^) the capacity fade was slightly more visible for LiTIM (61%) comparing to LiTFSI (53%). LiTDI electrolyte paired with Super P carbon performed the worse, comparing to other solutions. With 290 mAh g^–1^ of 290 mAh g^–1^, the fade was 62%.

For the Vulcan XC-72 (Figure 7), the best-performing electrolyte solution was the one containing LiTDI. Starting with an initial capacity of 455 mAh g^–1^, the capacity fade was 37%. The LiPDI solution performed slightly worse, starting with 425 mAh g^–1^, and the capacity fade was 49%. The performance of LiTFSI was comparable to the sample with the Super P electrode (290 mAh g^–1^, with a capacity fade of 39%). The LiTIM solution exhibited quite a capacity fade (65%) with an initial capacity of 330 mAh g^–1^. The less promising performance was presented by LiHDI, starting with 304 mAh g^–1^, but then fading to 110 mAh g^–1^ (64%).

Electrodes with Ketjen Black EC-600JD carbon (Figure 8) are presenting the most diverse performance. The LiHDI electrolyte solution reaches an initial capacity of 640 mAh g^–1^ but rapidly fades (55% compared to the first cycle). LiPDI, as previously noted, performed slightly worse, starting with 550 mAh g^–1^, and the capacity fade was 61%. Very promising capacities are exhibited by the solution of LiTDI, starting at 447 mAh g^–1^, with a capacity fade of 44%. The standard salt, LiTFSI, performed better compared to the Super P and Vulcan XC-72 samples, starting at 380 mAh g^–1^. By the time it reached the 100th cycle, the capacity fade was 44%. For the LiTIM salt, the performance was the least promising among all of the carbon blacks (220 mAh g^–1^ of initial capacity with a 50%).

As for the discharge curves, they are presented in Figures 6, 7, and 8 for cycles 1, 10, 20, 40, 60, 80, and 100. The tendencies are comparable to the discharge curves described in Section 3.2.1.

### 3.3 SEM and EDS Electrode Examination

SEM imaging highlights clear morphological distinctions among the three conductive carbon materials studied: Super P, Vulcan, and Ketjen Black, as can be seen in Figure 9. These differences reflect their manufacturing processes and directly influence their surface area, pore accessibility, and subsequent ability to host or interact with lithium salts.

**Figure 9:**
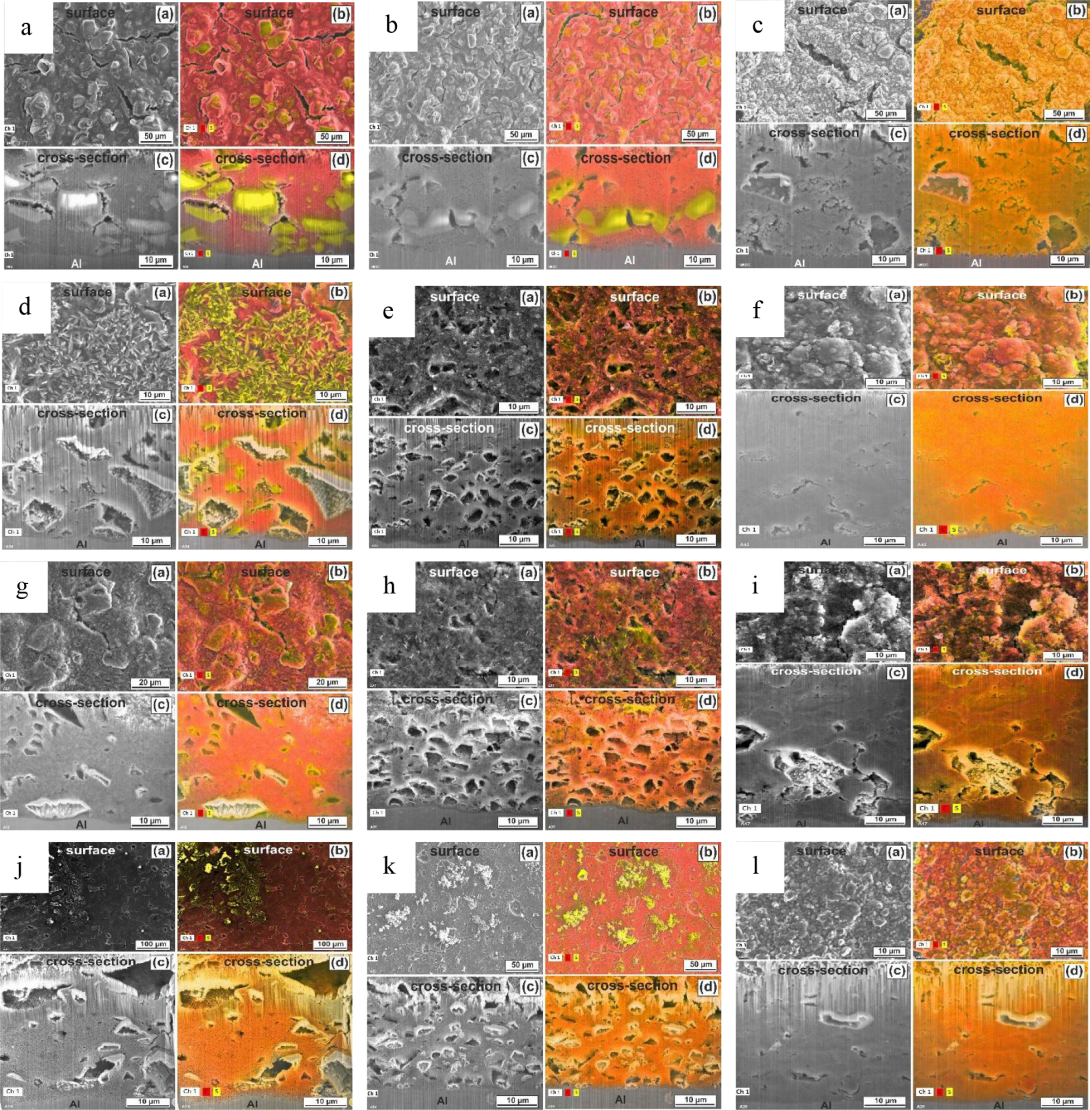
SEM and EDS imaging of the electrodes: (a) uncycled electrode with Super P, (b) uncycled electrode with Vulcan, (c) uncycled electrode with Ketjen Black, (d) cycled electrode with Super P and 1 M LiTFSI, (e) cycled electrode with Vulcan and 1 M LiTFSI, (f) cycled electrode with Ketjen Black and 1 M LiTFSI, (g) cycled electrode with Super P and 1 M LiTDI, (h) cycled electrode with Vulcan and 1 M LiTDI, (i) cycled electrode with Ketjen Black and 1 M LiTDI, (j) cycled electrode with Super P and 1 M LiTIM, (k) cycled electrode with Vulcan and 1 M LiTIM, and (l) cycled electrode with Ketjen Black and 1 M LiTIM.

Super P exhibits a uniform aggregate structure composed of nearly spherical primary carbon particles (20–50 nm). These particles are densely packed in secondary agglomerates, resulting in limited mesoporous volume and relatively low surface accessibility. The smoothness and uniformity of these aggregates make Super P a stable reference material, but also one that offers fewer active sites for interfacial salt deposition.

Vulcan, in contrast, presents more heterogeneity. Its aggregates are irregular, with rough surface protrusions and ill-defined pore entrances. This rough microtexture suggests the presence of surface functional groups and defect sites, which may influence the chemical interactions with electrolyte components. Nonetheless, its overall structure remains compact, offering a moderate increase in the accessible surface area relative to Super P.

Ketjen Black displays the most open and fractal morphology among the three. Its highly branched, chain-like carbon structures create extended pore networks and significant void space. The resulting high specific surface area (often >1400 m^2^ g^–1^ for Ketjen Black variants) is evident from the SEM images, where thin carbon filaments form multiscale porous frameworks. This topology is particularly favorable for uniformly hosting adsorbed or precipitated species due to the large interfacial volume.

Collectively, these morphological differences establish a framework for interpreting how each carbon type interacts with weakly coordinating lithium salts.

The introduction of lithium salt-based electrolytes—LiTFSI, LiTDI, and LiTIM—does not significantly alter the intrinsic microstructure of any of the carbon materials. Across all SEM images, the underlying carbon framework remains intact, confirming that salt addition does not cause collapse, swelling, or restructuring of carbon aggregates.

However, subtle contrast variations and additional bright features in the SEM micrographs revealed the presence of salt deposits. These features vary depending on the carbon type: 1) Super P: Salt deposits predominantly appear at particle junctions and within small interaggregate voids. The limited accessible surface leads to discrete, spatially separated salt accumulations. 2) Vulcan: The irregular surface provides more adsorption sites, and brighter features tend to form at surface asperities. While still moderately clustered, salt coverage is more heterogeneous and widespread than in Super P. 3) Ketjen Black: The large surface area and open structure promote a more uniform distribution. Salt accumulates not only on exposed branches but also deep within pore channels, suggesting capillary-driven infiltration during solvent evaporation. This leads to salt domains that appear to be more integrated into the carbon network. Across all systems, the absence of large crystalline domains suggests that the salts primarily form thin amorphous layers or microcrystalline clusters rather than bulk precipitates. This behavior aligns with the thermodynamic properties of weakly coordinating anions, which typically exhibit limited lattice energy and high solubility.

EDS spectra and maps confirm the presence and spatial distribution of salt-derived elements. Given EDS’s limitation in detecting lithium, the analysis centers on anion-specific elements: in the case of LiTFSI, we observed colocalized F and S, often appearing in sharply defined clusters. With LiTDI, widespread F and N signals, typically with fluorine more uniformly distributed, are clearly shown. LiTIM shows broad, diffuse N signals consistent with thin-layer deposition.

For Super P and Vulcan, EDS maps show localized enrichment corresponding to the small bright domains observed in the SEM. These enriched regions align with areas where surface topology allows salt accumulation. In Ketjen Black, elemental maps exhibit more continuous distributions, reinforcing the interpretation of better dispersion and deeper penetration of salts into the porous network. In samples containing LiTFSI, S and F colocalization serves as a clear indicator of intact TFSI anions. For LiTDI and LiTIM, nitrogen is more broadly distributed, but background contributions from the carbon matrix must be considered due to spectral overlap. No evidence of anion decomposition (e.g., sulfur-free fluorine) is strongly indicated, although partial redistribution of nitrogen in LiTDI samples might suggest minor interactions with carbon surfaces.

A hierarchy emerges in how effectively the different carbon materials host and disperse the added salts. With Ketjen Black, it can be seen that there is almost homogeneous salt distribution, effective surface infiltration, and a likely formation of continuous or semicontinuous interfacial layers. Vulcan layers show a sort of intermediate behavior; e.g., salt prefers high-curvature or defect-rich regions, and Super P films present the least uniform distribution of salt, confined to discrete and isolated deposits.

This hierarchy correlates strongly with each carbon’s accessible surface area and pore structure. These factors are critical for applications such as lithium metal anodes, solid-state electrolytes, or high-loading composite electrodes, where interfacial ion dissociation and transport dominate performance. The results indicate that Ketjen Black-based systems may support more effective ion redistribution and more uniform electrochemical interfaces compared to Super P or Vulcan.

To conclude this section, SEM and EDS analyses provide a coherent picture of how weakly coordinating lithium salts interact with three widely used conductive carbons. Intrinsic morphologies differ significantly among Super P, Vulcan, and Ketjen Black, influencing their surface chemistries and interaction potentials. Salt addition does not alter the primary carbon structures, indicating physical adsorption or thin-film deposition rather than chemical restructuring. EDS confirms anion-specific deposition patterns, despite lithium remaining undetectable. Salt domains correspond to bright SEM contrast.

Furthermore, salt distribution strongly depends on the carbon topology. Ketjen Black supports the most uniform and penetrating salt dispersion, Vulcan provides moderate, heterogeneous distribution, and, finally, Super P shows localized, discrete salt accumulation.

These findings highlight the importance of carbon microstructure in governing electrolyte–carbon interfacial behavior, a factor with direct implications for high-performance Li-ion, Li-metal, and solid-state battery systems.

## 4 Role of Fluorine in the Electrolyte

A parameter that allows for easy comparison of different systems in the Li–S systems is sulfur utilization (SU). This parameter enables the observation of certain regularities. Regardless of the carbon material used, at low currents of C/20 and C/10, the highest sulfur utilization is achieved with LiTFSI-based electrolytes. The exception is the LiTIM-based electrolyte, which at C/20 achieves comparable values when using Super P and Vulcan carbons. At low currents, systems based on the remaining Huckel anions perform well but noticeably worse. The situation changes when switching to charge and discharge cycles at higher currents of C/5 and C/2. While at C/5 current, the difference in SU between LiTFSI-based electrolytes and LiHDI-, LiPDI-, and LiTDI-based electrolytes is up to several percent, favoring the latter, at C/2 current, LiHDI- and LiPDI-based systems exhibit SU twice as high as the LiTFSI electrolyte. Specifically, the best results were obtained for LiHDI and LiPDI electrolytes, while LiTDI systems were comparable to or slightly better than those with LiTFSI. The same patterns were observed during long-term cycling at C/5 current. At high currents, the poorest, but still acceptable, results were obtained for LiTIM-based electrolytes.

Figure 10 summarizes the sulfur utilization of the electrodes for all investigated electrolyte systems and carbon black types at different current rates, as well as during extended cycling over 100 discharge/charge cycles.

**Figure 10:**
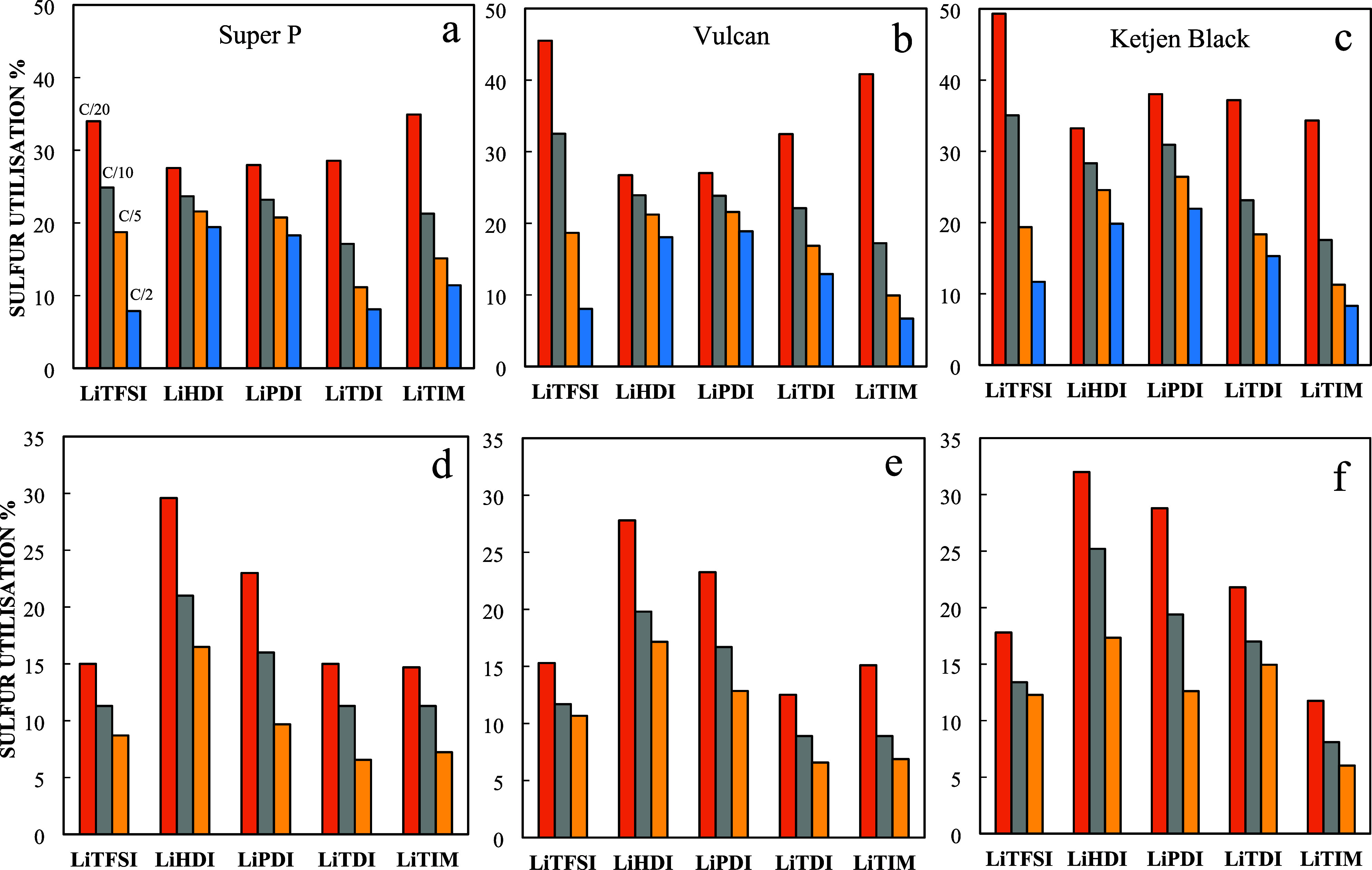
Graphical representation of the sulfur utilization for each carbon black and electrolyte solution. Figures (a), (b), and (c) represent the utilization during rate tests for currents C/20, C/10, C/5, and C/2. Figures (d), (e), and (f) represent sulfur utilization during long cycle tests (orange—after 10 cycles, gray—after 50 cycles, and yellow—after 100 cycles).

For the LiTFSI-based electrolyte, relatively high sulfur utilization is observed at low current densities (C/20 and C/10), particularly for electrodes containing Ketjen black. However, at higher current rates (C/5 and C/2), sulfur utilization decreases markedly. During the 100-cycle test, sulfur utilization for LiTFSI drops rapidly, reaching relatively low values after only 10 cycles compared to the other electrolytes, and it continues to decline after 50 and 100 cycles.

In contrast, electrolytes based on LiHDI and LiPDI exhibit significantly higher sulfur utilization at elevated current rates (C/5 and C/2) compared with all other electrolyte systems, irrespective of the carbon black employed. Moreover, during long-term cycling, these two electrolytes consistently demonstrate the highest sulfur utilization after 10, 50, and 100 cycles across all of the carbon black types.

For the LiTDI-based electrolyte, the sulfur utilization is strongly dependent on the type of carbon black used in the electrode. Electrodes incorporating Ketjen Black display rate performance comparable to that of LiPDI- and LiHDI-based systems and superior to that of LiTFSI. In contrast, Vulcan-based electrodes show slightly lower sulfur utilization, while Super-P-based electrodes exhibit performance comparable to or inferior to that observed with LiTFSI. During the 100-cycle test, electrodes containing Super P and Vulcan show moderate sulfur utilization, whereas those with Ketjen Black maintain high utilization levels, approaching those achieved with LiPDI and LiHDI electrolytes.

Behavior of LiHDI, LiPDI, and LiTDI salts in Li–S electrolytes tested on model electrodes stays in good agreement with our previous report.^53^

Finally, the performance of the nonfluorinated LiTIM electrolyte is considered. During rate capability testing, the highest sulfur utilization is obtained for electrodes based on Super P carbon black, while Vulcan- and Ketjen Black-based electrodes show utilization levels comparable to those of the LiTFSI electrolyte. In the 100-cycle test, a pronounced decrease in sulfur utilization is observed after 50 and 100 cycles for all electrode types, resulting in the lowest overall sulfur utilization among the investigated electrolyte systems.

Given the potential ecological advantages of the LiTIM-based electrolyte, further investigations were conducted to better understand its physicochemical properties. These included measurements of electrolyte ionic conductivity, scanning electron microscopy (SEM) analysis of electrode morphology, and Raman spectroscopy, which provided additional insights into the characteristics of this salt.

One of the explanations for the LiTIM electrochemical behavior could be ionic speciation, which is different from LiHDI, LiPDI, and LiTDI, as reported by us before.^46,53^

Starting from the relationship between ionic conductivity as a function of temperature for 1.0 M LiTIM salt concentration in DOL:DME (SI, Figure S4e) it is observed that as the temperature increases, the ionic conductivity increases as well. For all four Hückel salts used in the work, the observed trends in ionic conductivity follow the sequence σ_LiTDI_ > σ_LiPDI_ > σ_LiHDI_, σ_LiTIM_. At 20 °C, the values of ionic conductivity are as follows: 12 mS cm^–1^, 10 mS cm^–1^, 9 mS cm^–1^ ^54^, and 8 mS cm^–1^, respectively. The lower conductivity of LiTIM is closely related to its ionic association properties, which will be shown by local structure speciation in the following section.

Because of the difference in the chemical structure of LiTIM versus LiHDI, LiPDI, and LiTDI systems—presence of three nitrile groups instead of two and a fluorinated chain—changes in the Raman spectra (placed in the SI) of the studied solutions are visible. Raman spectra (Figure S4a, b, c and d) detect the evolution of the anion-related vibrational bands originating from imidazole ring vibrations, C–C imidazole stretching, C_2_–CN stretching, C–N imidazole symmetric stretching, and NCN in-plane vibrations located respectively at 2230, 2235, 1370, 1289, and 1010 cm^–1^ can be attributed to free ions—solvent-separated ionic pairs (SSIPs)—while at 2240, 2230, 1380, 1307, and 1020 cm^–1^ for contact ionic pairs (CIPs) with the cation linked to the imidazolium ring nitrogen.^55^ In comparison to LiHDI, LiPDI, and LiTDI, the spectrum of LiTIM shows complexity in the nitrile stretching vibration range and the presence of several overlapping bands originating from various ionic species, which makes the interpretation of the spectra difficult and allows only confirmation or contradiction of the participation of the nitrile group in the complex formation. According to the literature, where studies were based only on etheric solvents, only the δ_*NCN*_ vibration located at 1010 cm^–1^, gives a convenient probe to estimate the amount of the dissociated salt. In the case of use DOL–DME solvent mixture an overlapping of the solvent band with the band which is characteristic for the not dissociated salt at 1020 cm^–1^ is observed. The low intensity of this band and the relatively high noise-to-signal ratio make classical deconvolution highly risky. In this case, solvent spectra were subtracted from the spectra incorporating LiTIM salt, and the intensities of the 1010 cm^–1^ to 1020 cm^–1^ band were compared (Figure S4e,f). Such analysis is not quantitative but at least enables the explanation of observed trends with an increase in salt concentration. The ratios of the 1010 cm^–1^ to 1020 cm^–1^ band for 0.1 M, 0.3 M, 0.6 M, and 1.0 M samples are 0.5:1, 1.3:1, 1.9:1, and 1.6:1, respectively. The trend is as follows: with increasing concentration the dissociation of LiTIM increases up to the 0.6 M sample, reaches a maximum and for higher concentrations, the dissociation decreases; however, the total amount of free ions remains at a high level. Additionally, the ionic association properties of the LiTIM-based electrolyte can be investigated by analyzing the bands characteristic of ion–solvent interactions—shifts in the vibrational bands associated with DOL and DME, e.g., C–O and C–C stretching modes. The vibrational mode of pure DOL at ∼940 cm^–1^ (C–O stretching mode) decreases in intensity but remains stable in position, with no new bands emerging, which indicates that DOL molecules do not significantly participate in the solvation structure of Li^+^ cations. The bands of neat DME (850 and 823 cm^–1^, the coupled C–O stretching (υ_(CO)_) and CH_2_ rocking (δ_(CH2)_) vibrations, Figure S4d in the LiTIM based electrolyte decrease in intensity, while an emerging band at 874 cm^–1^—the value of bound DME—increases with concentration. DME molecules preferentially coordinate Li^+^ cations. Furthermore, the number of coordinated solvent molecules, derived from the υ_(CO)_ + δ_(CH2)_ band intensities of bound and free DME using eq 1, decreases from 5.28 in the 0.1 M salt to 1.86 in the 1.0 M salt (Table 3). In comparison, for 1 M solutions of LiHDI, LiPDI, and LiTDI, this number was equal to 2.6, 2.6, and 2.2, respectively, which means that in the LiTIM-based electrolyte, the concentration of free Li^+^ is lower as well as the dissociation degree, which is in good agreement with 1010 cm^–1^ band analysis. This is the main reason for the observed weaker electrochemical behavior versus the rest of the investigated Hückel salts. To support the above statements, the dependence of the conductivity of LiTIM salt versus concentration was recorded (Figure S4g). It is clearly seen that with increasing molar concentration, conductivity also increases up to the 0.6 M sample and then reaches a plateau. For the 1.0 M sample, conductivity is only slightly higher than for the 0.6 M, which is in correlation with Raman spectra analysis.

**Table 3:** Band Intensities if Bound and Free DME Solvent for Different LiTIM Solution Concentrations

Concentration/M	N	Li:DME Molar Ratio	Li:DOL Molar Ratio
0.1	5.28	1:44.4	1:71.5
0.3	3.66	1:14.8	1:23.8
0.6	2.24	1:7.4	1:11.9
1.0	1.86	1:4.4	1:7.2

## 5 Conclusions

This study systematically investigated key optimization strategies for lithium–sulfur (Li–S) batteries, emphasizing cathode engineering through tailored electrode compositions and novel electrolyte formulations with Hückel anion-based lithium salts. The strategic selection of conductive additives, binders, sulfur loadings, and advanced lithium salts yielded significant improvements in high-rate sulfur utilization, cyclability, and Coulombic efficiency.

A critical aspect of cathode optimization is the selection of conductive carbon additives. Three distinct carbon blacks were evaluated: Super P (Imerys), Vulcan XC-72 (Timcal), and Ketjen Black EC-600-JD. Among these, Ketjen Black delivered superior electrochemical performance, characterized by enhanced capacity retention and rate capability. This outcome aligns with its exceptionally high specific surface area, which facilitates superior sulfur dispersion, improved electronic conductivity, and better accommodation of volume changes during cycling. In contrast, Super P and Vulcan XC-72, with lower surface areas, exhibited diminished performance under high C rates, highlighting the pivotal role of carbon porosity and surface chemistry in maximizing sulfur utilization.

The electrolyte-to-sulfur (E/S) ratio has emerged as another vital parameter influencing cell performance. Optimal E/S values have been found to vary depending on the electrolyte solvent system and the lithium salt employed. Lower E/S ratios enhance the energy density but demand precise matching with salt dissociation efficiency and polysulfide solubility characteristics. This electrolyte-specific optimization is essential for lean-electrolyte Li–S systems, enabling high practical energy densities while mitigating the shuttle effect.

Electrolyte salt selection proved transformative, with Hückel anion-based salts outperforming conventional LiTFSI in several metrics. LiTIM, the fluorine-free variant, yielded the lowest performance among the novel salts due to suboptimal dissociation in DOL/DME solvents, though its results remained competitive and environmentally advantageous. Superior outcomes were achieved with LiPDI and LiHDI, which demonstrated exceptional high-rate sulfur utilization, stability, and efficiency-attributable to their tailored anion structures that suppress polysulfide dissolution more effectively than fluorinated benchmarks. LiTDI also performed robustly, positioning these salts as strong candidates to replace LiTFSI, particularly given the latter’s high fluorine content and associated environmental concerns.

Postmortem SEM analysis provided morphological insights, revealing no discernible differences attributable to electrolyte variations across samples. Instead, clear distinctions emerged among carbon black types. These observations reinforce the dominance of the carbon architecture in dictating long-term electrode integrity.

We firmly believe that our proposed electrolyte systems are fully compatible with the higher sulfur loadings required for practical, high-energy-density cells. Since LiTFSI is the current industry and academic standard for lithium–sulfur batteries, and our results demonstrate that Hückel-type salts perform at a comparable or even superior level under standardized conditions, there is no fundamental barrier to their application in high-loading electrode configurations. The transition to higher sulfur loadings primarily shifts the demand toward better electrolyte penetration and polysulfide management, meaning the areas where our fluorine-free mixtures have shown great promise. Therefore, the findings of this study provide a robust foundation for future scaling, and we are confident that these electrolytes will remain stable and efficient in practical cells with increased active material content.

This research demonstrates that optimal Li–S battery performance requires the simultaneous adjustment and holistic evaluation of multiple interdependent parameters. Modifying a single parameter in isolation, without considering the interplay with others, typically yields only mediocre results. These findings highlight the value of synergistic material design in bridging the gap between the theoretical potential and practical Li–S battery applications.

## Supplementary Material



## Notes

Optimization of the cathode process (homogeneity, performance tests, effect of carbon-to-sulfur ratio, and carbon porosity impact), as well as the Raman spectra and the conductivities of the LiTIM electrolyte (PDF)

## References

[1] Njema G. G., Ouma R., Kibet J. K., Kibet J. K. (2024). A Review on the
Recent Advances in Battery Development and Energy Storage Technologies. J. Renewable Energy.

[2] Broussely M., Archdale G. (2004). Li-Ion Batteries and Portable Power Source Prospects
for the next 5–10 Years. J. Power Sources.

[3] Liang Y., Zhao C. Z., Yuan H., Chen Y., Zhang W., Huang J. Q., Yu D., Liu Y., Titirici M. M., Chueh Y. L., Yu H., Zhang Q. (2019). A Review of Rechargeable
Batteries for Portable Electronic Devices. InfoMat.

[4] Zhao M., Li B. Q., Peng H. J., Yuan H., Wei J. Y., Huang J. Q. (2020). Lithium–Sulfur
Batteries under Lean Electrolyte
Conditions: Challenges and Opportunities. Angew.
Chem., Int. Ed..

[5] Fang R., Zhao S., Sun Z., Wang D. W., Cheng H. M., Li F. (2017). More Reliable Lithium-Sulfur
Batteries: Status, Solutions and Prospects. Adv. Mater..

[6] Li T., Bai X., Gulzar U., Bai Y. J., Capiglia C., Deng W., Zhou X., Liu Z., Feng Z., Proietti
Zaccaria R. (2019). A Comprehensive Understanding of Lithium–Sulfur
Battery Technology. Adv. Funct. Mater..

[7] Manthiram A., Fu Y., Su Y.-S. (2013). Challenges
and Prospects of Lithium–Sulfur Batteries. Acc. Chem. Res..

[8] Barke A., Cistjakov W., Steckermeier D., Thies C., Popien J. L., Michalowski P., Pinheiro Melo S., Cerdas F., Herrmann C., Krewer U., Kwade A., Spengler T. S. (2023). Green Batteries
for Clean Skies: Sustainability Assessment of Lithium-Sulfur All-Solid-State
Batteries for Electric Aircraft. J. Ind. Ecol..

[9] Benveniste G., Sánchez A., Rallo H., Corchero C., Amante B. (2022). Comparative
Life Cycle Assessment of Li-Sulphur and Li-Ion Batteries for Electric
Vehicles. Resour. Conserv. Recycl. Adv..

[10] Hong X., Mei J., Wen L., Tong Y., Vasileff A. J., Wang L., Liang J., Sun Z., Dou S. X. (2019). Nonlithium Metal–Sulfur
Batteries: Steps Toward a Leap. Adv. Mater..

[11] Steudel, R. Elemental Sulfur and Sulfur-Rich Compounds II; Springer, 2004; Vol.: 2, p. 248.

[12] Fedyaeva M., Lepeshkin S., Oganov A. R. (2023). Stability of Sulfur Molecules and
Insights into Sulfur Allotropy. Phys. Chem.
Chem. Phys..

[13] Zhou L., Danilov D. L., Qiao F., Wang J., Li H., Eichel R. A., Notten P. H. L. (2022). Sulfur Reduction Reaction in Lithium–Sulfur
Batteries: Mechanisms, Catalysts, and Characterization. Adv. Energy Mater..

[14] Yuan H., Peng H.-J., Huang J.-Q., Zhang Q. (2019). Sulfur Redox Reactions
at Working Interfaces in Lithium–Sulfur Batteries: A Perspective. Adv. Mater. Interfaces.

[15] Wang L., Zhang T., Yang S., Cheng F., Liang J., Chen J. (2013). A Quantum-Chemical Study on the Discharge Reaction Mechanism of Lithium-Sulfur
Batteries. J. Energy Chem..

[16] Wang J., Wang H., Jia S., Zhao Q., Zheng Q., Ma Y., Ma T., Li X. (2023). Recent Advances in Inhibiting Shuttle
Effect of Polysulfide in Lithium-Sulfur Batteries. J. Energy Storage.

[17] Li J., Gao L., Pan F., Gong C., Sun L., Gao H., Zhang J., Zhao Y., Wang G., Liu H. (2024). Engineering
Strategies for Suppressing the Shuttle Effect in Lithium–Sulfur
Batteries. Nano Micro Lett..

[18] Hofmann A. F., Fronczek D. N., Bessler W. G. (2014). Mechanistic
Modeling of Polysulfide
Shuttle and Capacity Loss in Lithium–Sulfur Batteries. J. Power Sources.

[19] Song M.-K., Zhang Y., Cairns E. J. (2013). A Long-Life,
High-Rate Lithium/Sulfur
Cell: A Multifaceted Approach to Enhancing Cell Performance. Nano Lett..

[20] Noh H., Song J., Park J. K., Kim H. T. (2015). A New Insight on
Capacity Fading of Lithium–Sulfur Batteries: The Effect of
Li2S Phase Structure. J. Power Sources.

[21] Knap V., Stroe D.-I., Swierczynski M., Teodorescu R., Schaltz E. (2016). Investigation of the Self-Discharge
Behavior of Lithium-Sulfur
Batteries. J. Electrochem. Soc..

[22] Wang J., Han W.-Q. (2022). A Review of Heteroatom Doped Materials for Advanced
Lithium–Sulfur Batteries. Adv. Funct.
Mater..

[23] Waluś S., Offer G., Hunt I., Patel Y., Stockley T., Williams J., Purkayastha R. (2018). Volumetric
Expansion of Lithium-Sulfur
Cell during Operation – Fundamental Insight into Applicable
Characteristics. Energy Storage Mater..

[24] Wang R., Yang J., Chen X., Zhao Y., Zhao W., Qian G., Li S., Xiao Y., Chen H., Ye Y. (2020). Highly
Dispersed Cobalt Clusters in Nitrogen-Doped
Porous Carbon Enable Multiple Effects for High-Performance Li–S
Battery. Adv. Energy Mater..

[25] Liu P., Wang Y., Liu J. (2019). Biomass-Derived Porous Carbon Materials
for Advanced Lithium Sulfur Batteries. J. Energy
Chem..

[26] Chen K., Sun Z., Fang R., Shi Y., Cheng H.-M., Li F. (2018). Metal–Organic
Frameworks (MOFs)-Derived Nitrogen-Doped Porous Carbon Anchored on
Graphene with Multifunctional Effects for Lithium–Sulfur Batteries. Adv. Funct. Mater..

[27] Fang R., Chen K., Yin L., Sun Z., Li F., Cheng H.-M. (2019). The Regulating Role of Carbon Nanotubes and Graphene
in Lithium-Ion and Lithium–Sulfur Batteries. Adv. Mater..

[28] Chen X., Zhao C., Yang K., Sun S., Bi J., Zhu N., Cai Q., Wang J., Yan W. (2023). Conducting Polymers
Meet Lithium–Sulfur Batteries: Progress, Challenges, and Perspectives. Energy Environ. Mater..

[29] Tao X., Yang Z., Yan R., Cheng M., Ma T., Cao S., Bai M., Ran F., Cheng C., Yang W. (2023). Engineering
MOFs-Derived Nanoarchitectures with Efficient Polysulfides Catalytic
Sites for Advanced Li–S Batteries. Adv.
Mater. Technol..

[30] Zheng J., Tian J., Wu D., Gu M., Xu W., Wang C., Gao F., Engelhard M. H., Zhang J. G., Liu J., Xiao J. (2014). Lewis Acid-Base Interactions
between Polysulfides and Metal Organic Framework in Lithium Sulfur
Batteries. Nano Lett..

[31] Deng S., Guo T., Heier J., Zhang C. (2023). Unraveling Polysulfide’s Adsorption
and Electrocatalytic Conversion on Metal Oxides for Li-S Batteries. Adv. Sci..

[32] Zhou Z. Q., Wang H. M., Yang L. B., Ma C., Wang J. T., Qiao W. M., Ling L. C. (2024). A Review of the Use of Metal Oxide/Carbon
Composite Materials to Inhibit the Shuttle Effect in Lithium-Sulfur
Batteries. New Carbon Mater..

[33] Li H., Wang X., Ma H., Guo D., Wu L., Jin H., Chen X., Wang S. (2024). A Review on
Catalytic Progress of
Polysulfide Redox Reactions on Transition Metal Sulfides in Li-S Batteries
from Structural Perspective. ChemElectroChem.

[34] Kaiser M. R., Han Z., Liang J., Dou S. X., Wang J. (2019). Lithium Sulfide-Based
Cathode for Lithium-Ion/Sulfur Battery: Recent Progress and Challenges. Energy Storage Mater..

[35] Duan L., Zhao L., Cong H., Zhang X., Lü W., Xue C. (2019). Plasma Treatment for Nitrogen-Doped
3D Graphene Framework by a Conductive
Matrix with Sulfur for High-Performance Li–S Batteries. Small.

[36] Zhang L., Liu D., Muhammad Z., Wan F., Xie W., Wang Y., Song L., Niu Z., Chen J. (2019). Single Nickel Atoms
on Nitrogen-Doped Graphene Enabling Enhanced Kinetics of Lithium–Sulfur
Batteries. Adv. Mater..

[37] Pang Q., Liang X., Kwok C. Y., Nazar L. F. (2015). Review—The
Importance of Chemical Interactions between Sulfur Host Materials
and Lithium Polysulfides for Advanced Lithium-Sulfur Batteries. J. Electrochem. Soc..

[38] Batyrgali N., Yerkinbekova Y., Tolganbek N., Kalybekkyzy S., Bakenov Z., Mentbayeva A. (2023). Recent Advances on Modification of
Separator for Li/S Batteries. ACS Appl. Energy
Mater..

[39] Li C., Liu R., Xiao Y., Cao F., Zhang H. (2021). Recent Progress of
Separators in Lithium-Sulfur Batteries. Energy
Storage Mater..

[40] Raza H., Bai S., Cheng J., Majumder S., Zhu H., Liu Q., Zheng G., Li X., Chen G. (2023). Li-S Batteries:
Challenges,
Achievements and Opportunities. Electrochem.
Energy Rev..

[41] Fransson L., Eriksson T., Edström K., Gustafsson T., Thomas J. O. (2001). Influence of Carbon Black and Binder
on Li-Ion Batteries. J. Power Sources.

[42] Cai W., Song Y., Fang Y., Wang W., Yu S., Ao H., Zhu Y., Qian Y. (2020). Defect Engineering on Carbon Black
for Accelerated Li-S Chemistry. Nano Res..

[43] Liu M., Deng N., Ju J., Fan L., Wang L., Li Z., Zhao H., Yang G., Kang W., Yan J. (2019). A Review: Electrospun
Nanofiber Materials for Lithium-Sulfur Batteries. Adv. Funct. Mater..

[44] Cheng J., Liu Y., Zhang X., Miao X., Chen Y., Chen S., Lin J., Zhang Y. (2021). Structure Engineering in Interconnected Porous Hollow
Carbon Spheres with Superior Rate Capability for Supercapacitors and
Lithium-Sulfur Batteries. Chem. Eng. J..

[45] Zhang Y., Gao Z., Song N., He J., Li X. (2018). Graphene and Its Derivatives
in Lithium–Sulfur Batteries. Mater. Today
Energy.

[46] Kottarathil A., Slim Z., Ishfaq H. A., Jeschke S., Żukowska G. Z., Marczewski M., Lech K., Johansson P., Wieczorek W. (2024). The Role of the Anion in Concentrated Electrolytes
for Lithium-Sulfur Batteries. J. Electrochem.
Soc..

[47] Mao Y., Li T., Abuelgasim S., Hao X., Xiao Y., Li C., Wang W., Li Y., Bao E. (2024). Systematic Insight
of the Behavior of LiNO3 Additive in LiS Batteries with Gradient S
Loading. J. Energy Storage.

[48] Park C., Ronneburg A., Risse S., Ballauff M., Kanduč M., Dzubiella J. (2019). Structural and Transport Properties
of Li/S Battery
Electrolytes: Role of the Polysulfide Species. J. Phys. Chem. C.

[49] Jin T., Li X. Y., Zhao M., Feng S., Li Z., Chen Z. X., Peng H. J., Li B. Q., Huang J. Q. (2025). Promoting
the Rate Performances of Weakly Solvating Electrolyte-Based Lithium–Sulfur
Batteries. Angew. Chem., Int. Ed..

[50] Jin T., Zhao M., Li X.-Y., Chen Z.-X., Li B.-Q., Huang J.-Q., Zhang Q. (2026). Reducing Polysulfide
Hydrodynamic
Radius toward Low-Temperature Lithium–Sulfur Batteries. Chem.

[51] Li Z., Li B.-Q., Chen L.-L., Gao Y.-C., Bi C.-X., Zhao M., Chen X., Li X.-Y., Zhang Q. (2026). Regulating
Lithium Bond to Reduce Polysulfide Parasitic Reactivity for High-Stability
Lithium Metal Anode. Angew. Chem..

[52] Armand M., Johansson P., Bukowska M., Szczeciński P., Niedzicki L., Marcinek M., Dranka M., Zachara J., Żukowska G., Marczewski M., Schmidt G., Wieczorek W. (2020). Review—Development
of Hückel Type Anions: From Molecular Modeling to Industrial
Commercialization. A Success Story. J. Electrochem.
Soc..

[53] Kottarathil A., Luong N. T., Cardona C. C., Jeschke S., Hosaka T., Żukowska G. Z., Marczewski M., Wieczorek W., Johansson P. (2025). Hückel Anion Based Concentrated Electrolytes
for Lithium–Sulfur Batteries. Phys. Chem.
Chem. Phys..

[54] Dąbrowska A. T., Izdebska N., Żero E., Smolarek M., Jastrzębski D., Jastrzębski C., Żukowska G. Z., Jankowski P., Siekierski M., Piszcz M. (2025). Corrosion Investigation of Current
Collector in Solid State Lithium Sulphur Batteries. Appl. Phys. A.

[55] Żukowska G. Z., Piszcz M., Gańko K., Więckowski M., Królikowski M., Poterała M., Dranka M. (2024). Basic Properties of
Glyme-Based Electrolytes Doped with Lithium 2,4,5-Tricyanoimidazolide
(LiTIM). Mater. Chem. Phys..

[56] Niedzicki L., Zukowska G. Z., Bukowska M., Szczeciński P., Grugeon S., Laruelle S., Armand M., Panero S., Scrosati B., Marcinek M., Wieczorek W. (2010). New Type of
Imidazole Based Salts Designed Specifically for Lithium Ion Batteries. Electrochim. Acta.

[57] Seteiz K., Häberlein J. N., Heizmann P. A., Disch J., Vierrath S. (2023). Carbon Black
Supported Ag Nanoparticles in Zero-Gap CO 2 Electrolysis to CO Enabling
High Mass Activity. RSC Adv..

[58] Smoliński M., Szczęsna-Chrzan A., Trzeciak T., Ossowska A., del Campo E., Żukowska G. Z., Żero E., Zybert M., Ronduda H., Ostrowski A. (2025). From by-product of the petrol gasification
to carbon black in lithium-ion
batteries – Novel electron-conductive agents. Appl. Phys. A.

[59] Amin A. S., Caidi A., Lange T., Radev I., Sandbeck D. J. S., Philippi W., Kräenbring M.
A., Öztürk M., Peinecke V., Lerche D., Özcan F., Segets D. (2025). Key Control Characteristics of Carbon Black Materials
for Fuel Cells and Batteries for a Standardized Characterization of
Surface Properties. Part. Part. Syst. Charact..

