# Supplementary material for: From Fluorinated to All-Fluorine-Free Systems: Hückel Anion-Based Electrolytes for Lithium–Sulfur Batteries with Enhanced High C‑Rate Sulfur Utilization
Source: ACS Omega. 2026 Jun 17;11(25):37023–38. doi: 10.1021/acsomega.6c01071 (PMC13325106; doi:10.1021/acsomega.6c01071)
Supplement: Supplementary file 1 [file ao6c01071_si_001.pdf]

From Fluorinated to All Fluorine Free systems - Hückel Anion based Electrolytes for Lithium-Sulfur Batteries with Enhanced High C-Rate Sulfur Utilization

Maciej Smoliński [a,], Adam Łaszcz [b], Aleksandra Ossowska [a], Marek Marcinek [a] and Maciej Marczewski [a,*]*

[*]Corresponding author: Maciej Smoliński, maciej.smolinski.dokt@pw.edu.pl, Maciej Marczewski, maciej.marczewski@pw.edu.pl

[a] Warsaw University of Technology, Faculty of Chemistry, Noakowskiego 3, 00-664 Warsaw, Poland

[b]Łukasiewicz Research Network, Institute of Microelectronics and Photonics, al. Lotników 32/46, 02-668 Warsaw, Poland

Supporting Information

1. Optimization of the cathode

1.1 Homogeneity examination

In order to make this research, as a first step the optimization of the electrode's composition was carried out. The optimization concerned only the cells with 1M LiTFSI electrolyte, as in this step the work was focused on setting up the most efficient sulfur to carbon ratio and comparing the carbon black's conductivity. Also, the aspect of binder was considered, such as sodium carboxymethyl cellulose's medium chain weight and concentration of the solution are the parameters from which the rheology and homogenization of the electrode slurry depends. To make the optimization, all the electrodes shown in Table S1 were made (extended by applying different NaCMC water solution concentration). The first step was to compare the electrode layers visually (Figure S1), which concerned the eventual appearance of cracks, agglomerates, or layer-to-aluminum adhesion problems. If the electrode had problems with one or few of listed parameters, the layer was prepared again in order to make sure it's repetitive. Figure 1 show the example of a homogenous, well-coated electrode layer (a) and the layer with visible surface cracks (b).

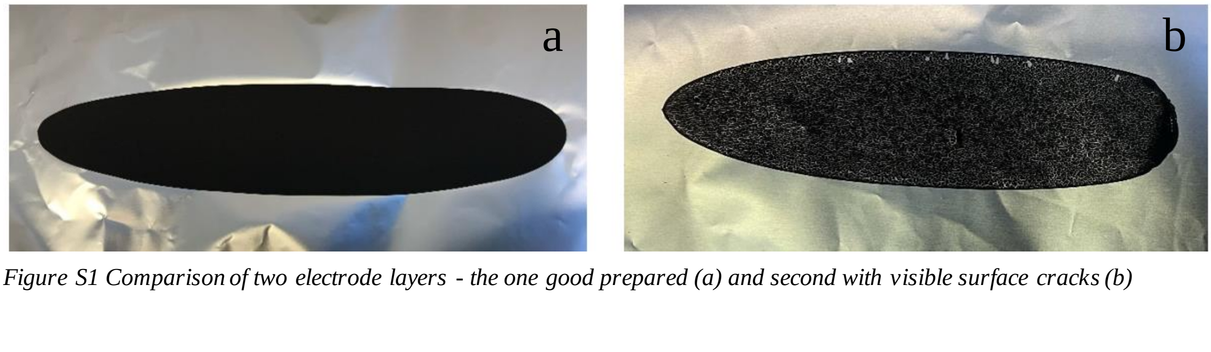

Figure S1 Comparison of two electrode layers - the one good prepared (a) and second with visible surface cracks (b)

1.2 Performance test

Once the electrode layers have been examined visually, the second step of the optimization was to compare the electrochemical performance of the electrodes containing different compositions. Cells

preparation and type of electrochemical tests were described in sections 2.3 and 2.4. Results are shown in Table S1. The values of the specific capacity for each current are the average from 3 consecutive cycles. As observed, it is rather difficult to compare the values of the specific capacities having more parameters in change. Ketjen Black EC-600JD presents very high values of specific capacity both for C/100 and C/2 currents (1279, 1059, 1055 and 870 mAh g^{-1} for C/100 and 184, 195, 180, 190 mAh g^{-1} for C/2). Vulcan XC-72 carbon black shows high values of the capacities in forming cycle C/100 (1183, 1047, 1026, 1179, 964 mAh g^{-1}), but the performance for faster charging (C/2) is rather poor 135, 108, 99, 114, 79 mAh g^{-1}). For the Super P carbon black, the initial capacities during the forming cycle are lower comparing to Vulcan XC-72 and Ketjen Black EC-600JD. At the same time for the current C/2 the values of the capacities are visibly higher comparing to Vulcan XC-72, but lower comparing to Ketjen Black EC-600-JD. In order to look deeper into the electrodes performance, more analysis of the results have been done, presented in the next sections.

Table S1 Capacity results of the optimized electrodes

Amount of sulfur	Carbon type	NaCMC Binder	Current						
			C/100	C/20	C/10	C/5	C/2	C/5	
60%	Super P	Mw=700 000, C=1.5%	967	597	495	264	123	209	Specific capacity [mAh g^{-1}]
		Mw=700 000, C= 2.0%	943	570	457	314	132	259	
		Mw=250 000, C=2.5%	1152	608	291	175	131	153	
	Ketjen black EC-600JD	Mw=700 000, C=1.5%	1279	776	536	310	184	251	
		Mw=700 000, C=2.0%	1059	823	585	323	195	281	
	Vulcan XC-72	Mw=700 000, C=1.5%	1183	763	545	313	135	263	
		Mw=700 000, C=2.0%	1047	663	572	292	108	253	
		Mw=250 000, C=2.5%	1026	671	516	277	99	221	
70%	Super P	Mw=700 000, C=1.5%	791	465	348	220	115	166	
		Mw=700 000, C=2.0%	735	568	431	257	140	198	
		Mw=250 000, C=2.5%	1007	511	455	239	125	171	
		Mw=250 000, C=3.0%	899	518	337	214	152	180	
	EC-600JD	Mw=700 000, C=1.5%	1055	816	631	345	180	276	
		Mw=700 000, C=2.0%	870	626	460	294	190	239	
	Vulcan XC-72	Mw=700 000, C=1.5%	1179	332	312	217	114	182	
		Mw=700 000, C=2.0%	964	388	340	190	79	145	

1.3 Effect of carbon/sulfur ratio

After comparing the general values of the specific capacities of different cathodes, discharge curves profiles and repeatability of the following-cycles capacities were taken into the consideration. Sulfur/carbon ratio plays a critical role in determining the electrode's electrochemical performance.

Optimizing the sulfur/carbon ratio is therefore essential to ensure sufficient electron transport while maintaining a high active material loading and stable cycling performance. Figure S2a is presenting discharge curve profiles for the electrodes with 60% and 70% of Super P carbon black. As observed, they are differing to a minimal extent, mostly in the specific capacity. The voltages of the plateaus are almost identical. Figure S2b is presenting the comparison of the rate test. Despite the differences of the capacities, in favor of the electrode with 60% of sulfur content, the tendency repeatability of each of the 3 cycles per each current is the same.

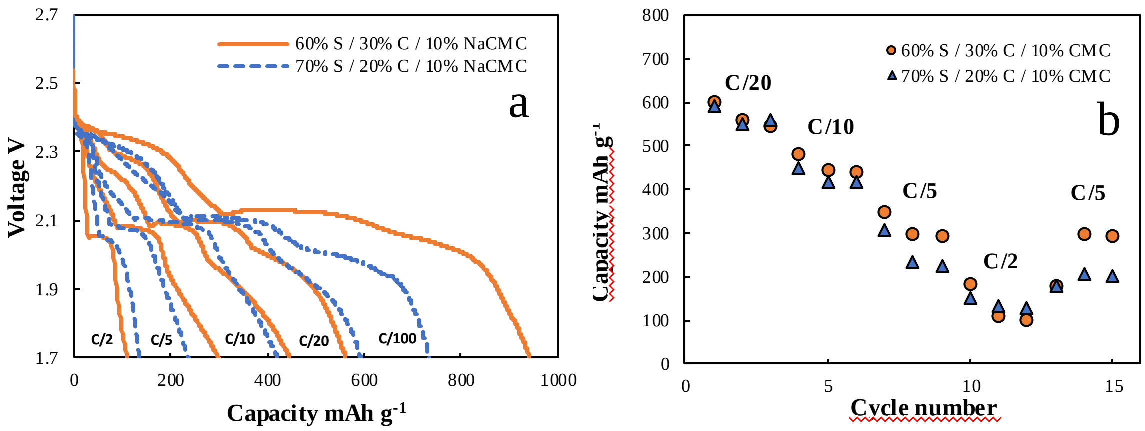

Figure S2 (a) discharge curves comparison for two types of the electrodes - 60 and 70% of sulfur loading with (b) rate test

1.4 Carbon porosity impact

Next parameter compared in the research was the conductive carbon additive. Carbon porosity plays a crucial role in the performance of lithium-sulfur batteries. A highly porous carbon matrix provides a larger surface area for sulfur loading and improves the electrical conductivity throughout the cathode. It also helps trap soluble lithium polysulfides, mitigating the shuttle effect and enhancing cycle life. Additionally, tailored porosity enables better electrolyte infiltration and ion transport, which contributes to higher sulfur utilization and overall battery efficiency. In the research three types of carbon blacks were used – Super P, Vulcan XC-72 and Ketjen Black EC-600JD. They are differing in the textural parameters. The specific surface areas are respectively: ~64 cm^2 g^{-1}, ~250 cm^2 g^{-1} and ~1100 cm^2 g^{-1} [50–52]. Figure S4 illustrates the electrochemical performance of electrodes incorporating different carbon blacks. Among the tested materials, Super P exhibited the lowest average capacities at both C/20 and C/10 rates (570 and 457 mAh g^{-1}, respectively), compared to Ketjen Black EC-600-JD, which delivered significantly higher capacities (823 and 585 mAh g^{-1}, respectively), and Vulcan XC-72, which also outperformed Super P (763 and 545 mAh g^{-1}, respectively). At a higher charge rate of C/2, Ketjen Black EC-600JD continued to demonstrate superior performance, achieving an average capacity of 195 mAh g^{-1}, whereas Vulcan XC-72 and Super P showed lower values of 135 and 132 mAh g^{-1}, respectively. The discharge plateaus shown on figure S4b differs significantly depending on the type of carbon black

used. All three samples exhibit the characteristic two-plateau discharge profile of Li-S batteries: the upper plateau (2.3 – 2.1 V), corresponding to the reduction of elemental sulfur to long-chain polysulfides (Li_2S_8 to Li_2S_4), and the lower plateau (2.1 – 1.9 V), associated with the formation of insoluble short-chain species (Li_2S_2 and Li_2S). Among the samples, the electrode containing Super P shows the most pronounced and extended plateaus, especially in the upper voltage region, indicating good electrochemical kinetics. Ketjen Black EC-600JD also demonstrates well-defined plateaus, although slightly shorter than those of Super P, suggesting slightly less efficient sulfur conversion despite its high surface area. In contrast, the electrode with Vulcan XC-72 exhibits a more sloped and compressed discharge profile, with both plateaus less clearly defined. This behavior suggests limited electron transport, even though the capacity value is higher comparing to Super P.

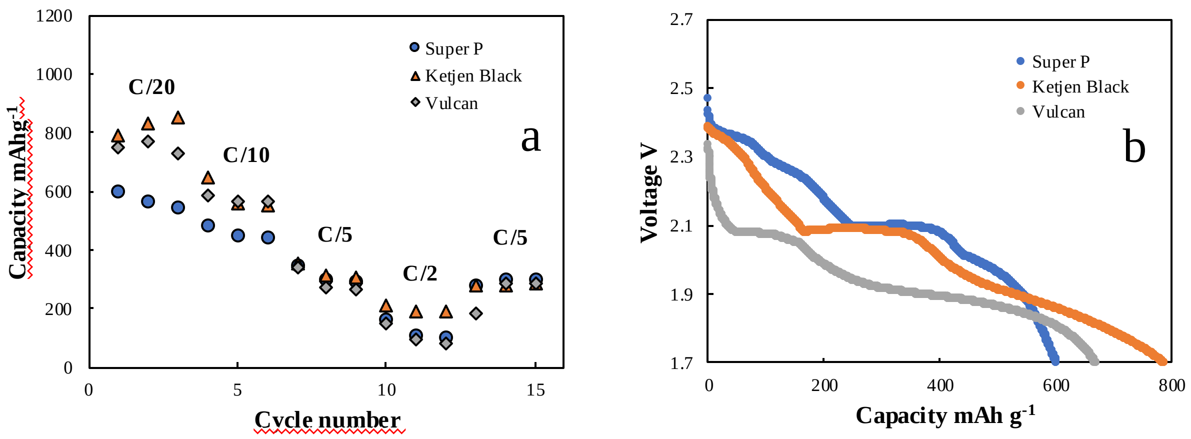

Figure S3 (a) rate test comparing the performance of different carbon blacks applied in the research together with (b) discharge curves.

2. Role of fluorine in the electrolyte

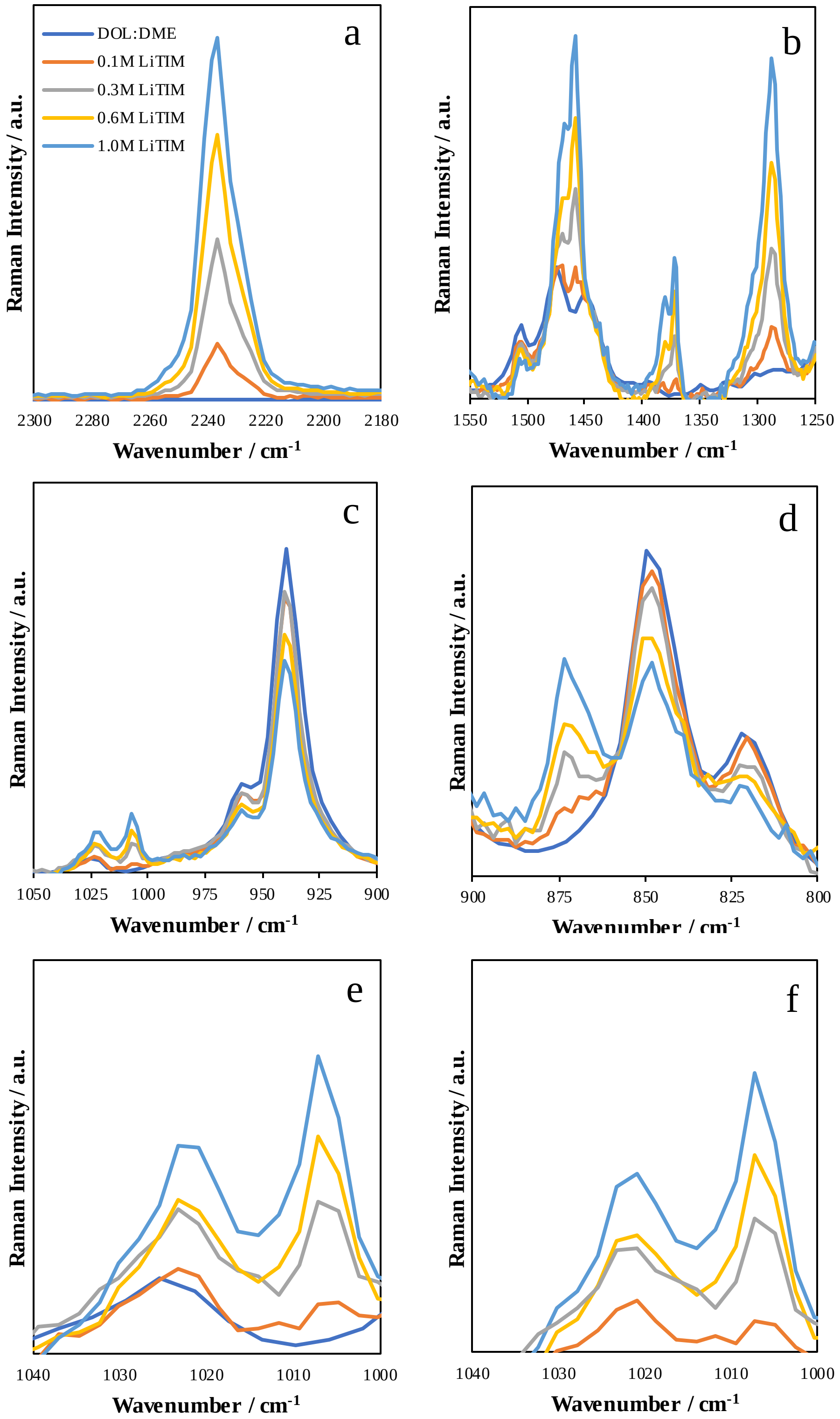

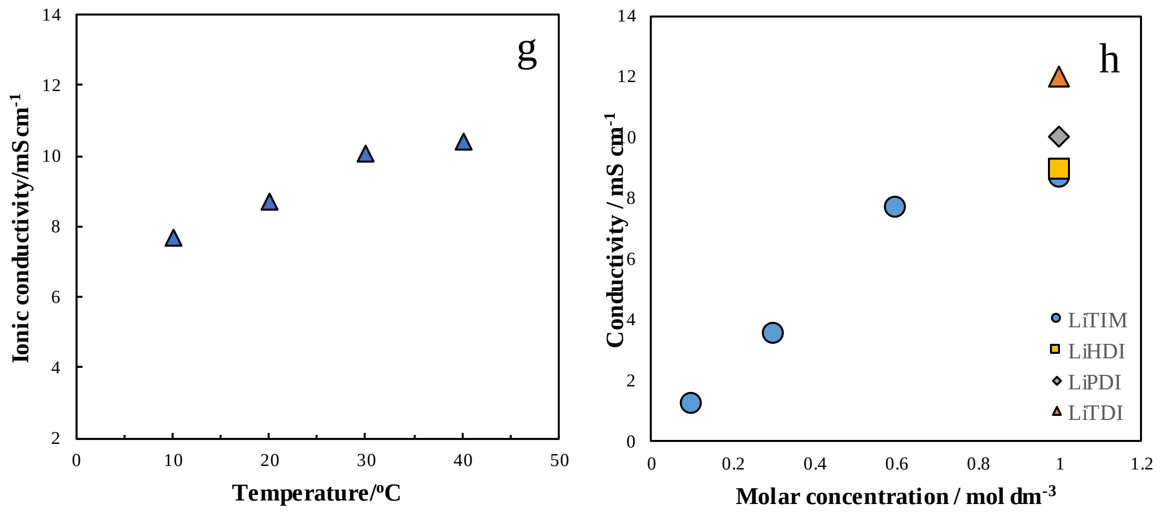

Figure S4 Raman spectra of LiTIM in DOL:DME: a – wavenumber 2300-2180 cm^{-1}, b – wavenumber 1550-1250 cm^{-1}, c – wavenumber 1050-900 cm^{-1}, d – wavenumber 900-800 cm^{-1}, e – wavenumber 1040-1000 cm^{-1}, f – wavenumber 1040-1000 cm^{-1} (Curves represent different salt concentrations: DOL:DME – blue, 0.1M – orange, 0.3M - gray, 0.6M – yellow; 1.0M – purple), g – *Ionic conductivity of LiTIM in DOL:DME, h – Ionic conductivity of LiTIM, LiTDI, LiPDI and LiHDI depending on the molar concentration for 20 ºC*
